# Optimizing the localization of astaxanthin enzymes for improved productivity

**DOI:** 10.1186/s13068-018-1270-1

**Published:** 2018-10-10

**Authors:** Lijun Ye, Xinna Zhu, Tao Wu, Wen Wang, Dongdong Zhao, Changhao Bi, Xueli Zhang

**Affiliations:** 1 Tianjin Institute of Industrial Biotechnology, Chinese Academy of Sciences, Tianjin, 300308 People’s Republic of China; 2 Key Laboratory of Systems Microbial Biotechnology, Chinese Academy of Sciences, Tianjin, 300308 People’s Republic of China

**Keywords:** Localization, Astaxanthin, β-Ionone, Carotenoids, *Escherichia coli*

## Abstract

**Background:**

One important metabolic engineering strategy is to localize the enzymes close to their substrates for improved catalytic efficiency. However, localization configurations become more complex the greater the number of enzymes and substrates is involved. Indeed, optimizing synthetic pathways by localizing multiple enzymes remains a challenge. Terpenes are one of the most valuable and abundant natural product groups. Phytoene, lycopene and β-carotene serve as common intermediates for the synthesis of many carotenoids and derivative compounds, which are hydrophobic long-chain terpenoids, insoluble in water and usually accumulate in membrane compartments.

**Results:**

While β-ionone synthesis by β-carotene cleavage dioxygenase PhCCD1 and astaxanthin synthesis by β-carotene ketolase (CrtW) and β-carotene hydroxylase (CrtZ) differ in complexity (single and multiple step pathways), the productivity of both pathways benefited from controlling enzyme localization to the *E. coli* cell membrane via a GlpF protein fusion. Especially, the astaxanthin synthesis pathway comprises both CrtW and CrtZ, which perform four interchangeable reactions initiated from β-carotene. Up to four localization strategies of CrtW and CrtZ were exhaustively discussed in this work, and the optimal positioning strategy was achieved. CrtW and CrtZ were linked using a flexible linker and localized to the membrane via a GlpF protein fusion. Enzymes in the optimal localization configuration allowed a 215.4% astaxanthin production increase.

**Conclusions:**

This work exploits a localization situation involving membrane-bound substrates, intermediates and multiple enzymes for the first time, and provides a workable positioning strategy to solve problems in similar circumstances.

**Electronic supplementary material:**

The online version of this article (10.1186/s13068-018-1270-1) contains supplementary material, which is available to authorized users.

## Background

**Fig. 1:**
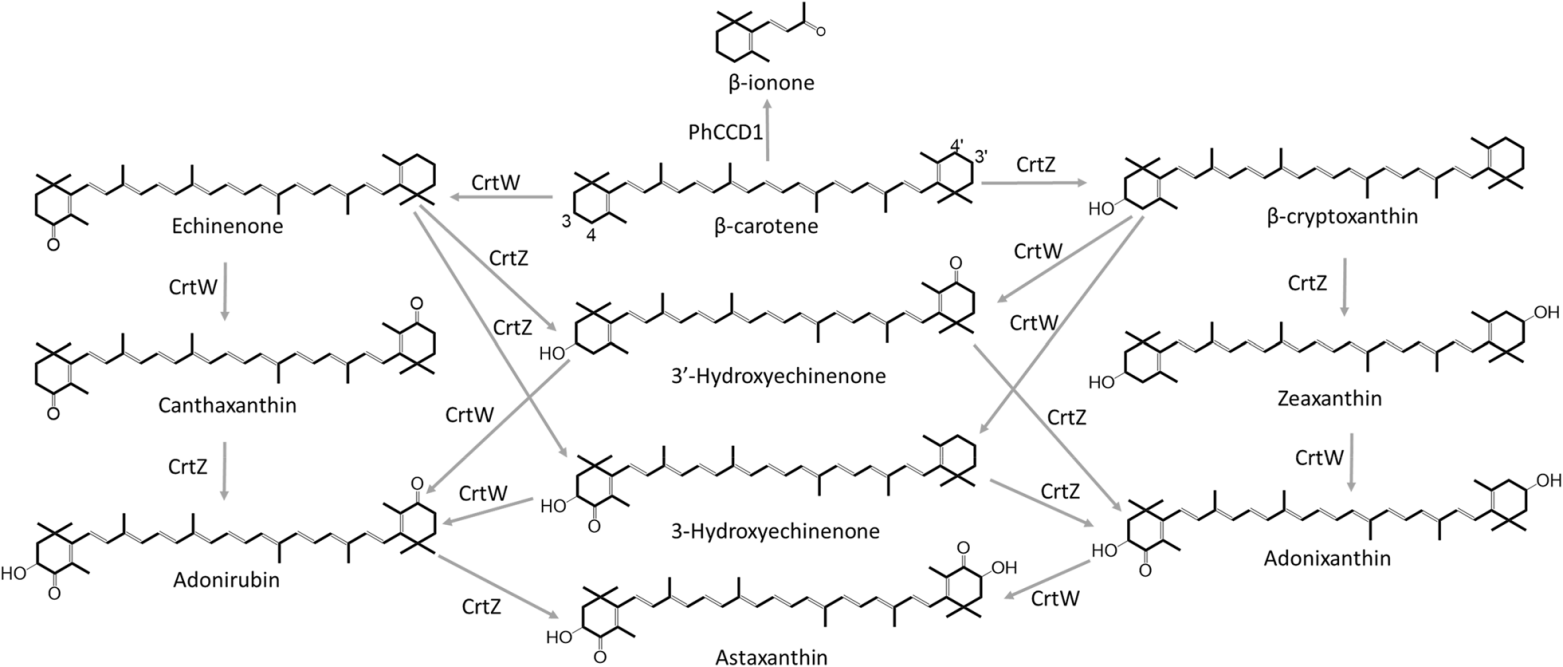
A schematic diagram of the astaxanthin and β-ionone synthesis pathways. The complex astaxanthin synthesis pathway comprises two enzymes, β-carotene ketolase (CrtW) and β-carotene hydroxylase (CrtZ), which perform four interchangeable reactions initiated from β-carotene, the substrate of the reaction

Terpenes, and especially the carotenoids, are one of the most valuable and abundant natural product groups [1, 2]. Metabolic engineering of microbial factories for the production of carotenoids has been a hotspot for decades, and numerous achievements have led to the advancement of this field [2, 3]. While many carotenoids and derivative compounds were produced heterologously, this remains challenging since the common intermediates phytoene, lycopene and β-carotene (Fig. 1) are hydrophobic long-chain terpenoids, insoluble in water, and usually accumulate in the membrane compartments [4–6]. Due to the specific localization of hydrophobic substrates, spatial optimization of their metabolism has attracted much attention in the metabolic engineering community, with some notable results. For example, an eight and 20-fold improvement in the production of valencene and amorphadiene was achieved by mitochondrial targeting of valencene and amorphadiene synthase in yeast, respectively [7]. Localizing the Ehrlich pathway into yeast mitochondria increased isobutanol production by 260%, whereas overexpression of the same pathway in the cytoplasm only improved yields by 10% [8]. Lv et al. proposed a dual metabolic engineering of cytoplasmic and mitochondrial acetyl-CoA utilization to boost isoprene synthesis in *S. cerevisiae*, which increased isoprene production 2.1- and 1.6-fold relative to the recombinant strains with solely mitochondrial or cytoplasmic engineering, respectively [9]. On the other hand, there were also excellent works concerning optimization of bacteria enzyme localization. For instance, using the protein scaffolds to recruit enzymes in a metabolic pathway, Dueber et al. acquired 77-fold improvement of product titer of mevalonate with low enzyme expression and reduced metabolic load [10]. Lee et al. exhibited the great potential of cytoscaffold for enzyme organization [11]. Furthermore, in addition to protein scaffolds, DNA scaffolds [12, 13] and lipid-containing scaffolds [14] are also applied to improve the pathway efficiency. In addition to scaffolds, enzymes were also located to bacterial microcompartments (MCPs), such as ethanolamine utilization (Eut) [15] and 1,2-propanediol utilization (Pdu) MCPs for similar purposes [16]. The key point of these strategies was to localize the enzymes as close as possible to their substrates. However, localization configurations become more complex when more enzymes and reaction steps are involved. Thus, optimizing synthetic pathways by positioning multiple enzymes remains a challenge.

**Fig. 2:**
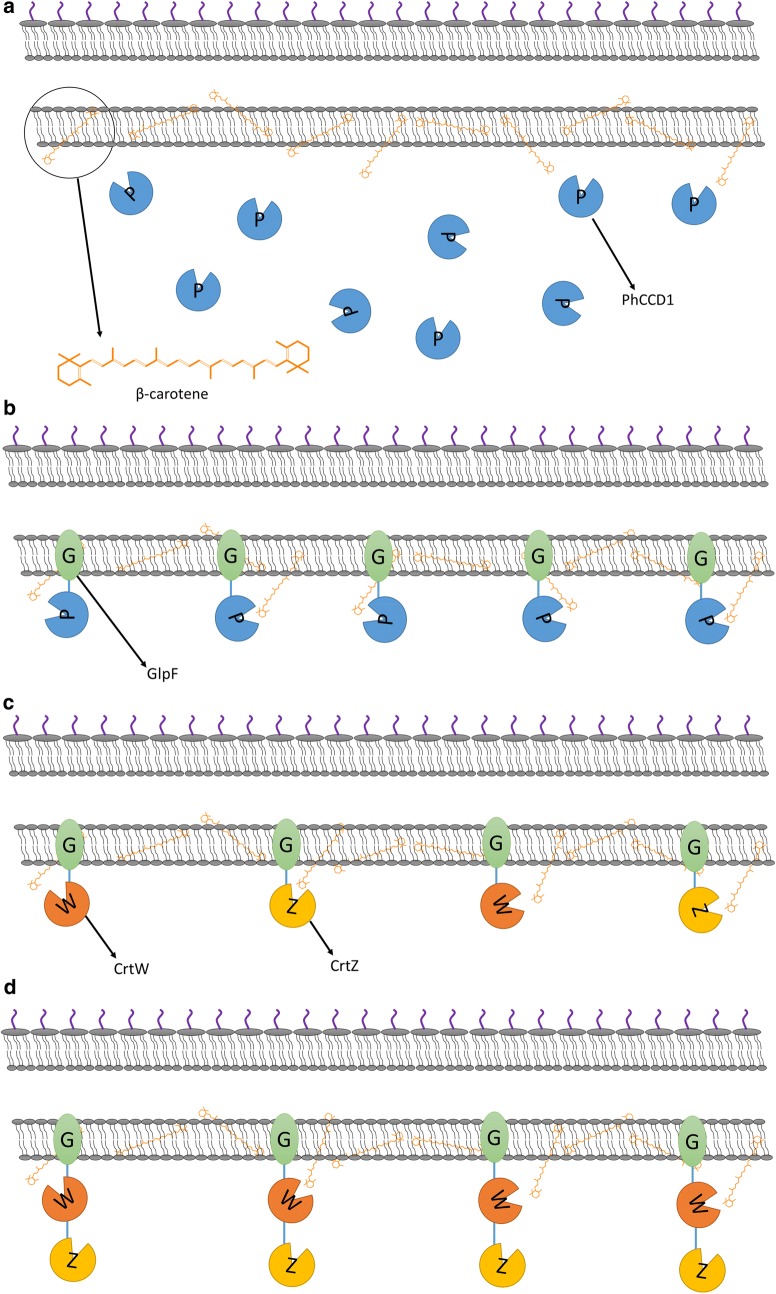
Enzyme locations in the host strain producing the β-carotene substrate. **a** Soluble cytoplasmic enzymes (PhCCD1) in the cells. **b** PhCCD1 localized to the membrane compartment by fusion with GlpF. **c** CrtW and CrtZ both localized to the membrane separately. **d** GlpF fused to the fusion protein CrtW-CrtZ to target it to the membrane

**Fig. 3:**
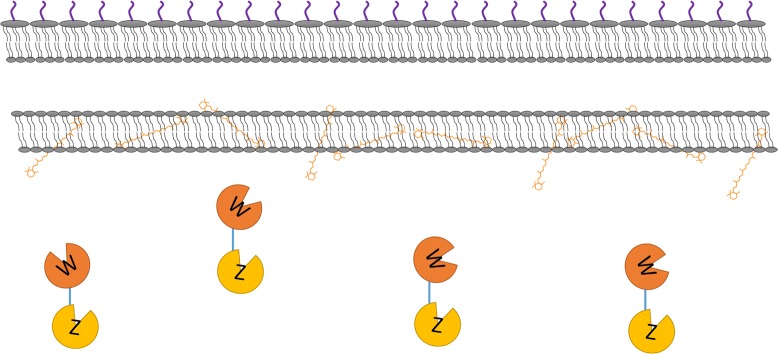
Enzyme locations in the host strain producing the β-carotene substrate. CrtW and CrtZ linked by a flexible eight-amino acid linker

The apocarotenoid β-ionone, which is derived from β-carotene, is notably valuable in the flavoring industry due to its characteristic rose-like aroma [17]. The carotenoid cleavage dioxygenase (CCD) PhCCD1 from *Petunia hybrida*, encoded by the *CCD1* gene (*PhCCD1*), was used for heterologous β-ionone production [18]. The CCD enzymes are usually cytoplasmic proteins [19, 20] and there was no predicted transmembrane region on PhCCD1 based on the information in ExPASy and UniProt [21]. Moreover, our previous work has shown that most β-carotene accumulated with cell membrane compartment in *E. coli* [22]. This means that it may be located far away from its substrate, the membrane-bound β-carotene, which would obviously decrease its catalytic efficiency. Thus, PhCCD1 can be localized to various compartments of the *E. coli* cell to investigate the relationship between the location of PhCCD1 and its catalytic efficiency (Figs. 2a, b, 3).

Astaxanthin is one of the strongest antioxidants in nature [1], and has a tremendous potential for applications in healthcare and pharmaceuticals [2, 23]. The heterologous synthesis pathway of astaxanthin comprises two enzymes, β-carotene ketolase (CrtW) from *Brevundimonas* sp. SD212 [24, 25] and β-carotene hydroxylase (CrtZ) from *Pantoea agglomerans* [26], which perform four interchangeable reactions initiated from β-carotene. Although CrtW and CrtZ might contain transmembrane regions according to the predicted structures from ExPASy and UniProt [21], there was no information regarding their original location, let alone their location in the heterologous hosts. Thus, the optimal localization of the astaxanthin synthesis pathway enzymes is a more complex problem than that of the single-step β-ionone synthesis pathway.

In this study, we applied *E. coli* localizing tags to position PhCCD1 into different cell compartments to investigate how the localization of the enzyme could affect its catalytic efficiency. Moreover, the localization situation of CrtW and CrtZ was studied to determine an optimal configuration for maximizing the production of astaxanthin.

## Methods

### Strains, media and growth conditions

The strains used in this study are listed in Table 1. During strain construction, cultures were grown aerobically at 37 °C in Lysogeny broth (per liter: 10 g Difco tryptone, 5 g Difco yeast extract and 5 g NaCl, referred to as LB in the following), or in LB with 2% glycerin for fermentation. For seed cultures, single colonies were picked from the plates and used to inoculate 15 × 100 mm tubes containing 4 mL of LB with 34 mg/L chloramphenicol, and grown at 37 °C and 250 rpm overnight. The resulting seed culture was subsequently used to inoculate a 100-mL flask containing 10 mL of fermentation medium with 34 mg/L chloramphenicol to an initial OD_600_ of 0.05, and grown at 30 °C and 250 rpm. After 48 h of growth, the cells were collected for the measurement of the products. IPTG was added after 6 h of inoculation to 0.1 mM final concentration when CAR025 was cultured. For β-ionone production, 1 mL dodecane was added to the cultures to capture this volatile product.

**Table 1:** Strains and plasmids in this study

Strains/plasmids	Relevant characteristics	Source/notes
Strains		
CAR010	CAR005 [27], mRSL-4::ispG, mRSL-14::ispH	Unpublished
CAR003	CAR010, ∆*crtX*	Unpublished
CAR025	CAR005 [27], ispG-mRSL-4, ispH-mRSL-14, replacing the promoter of *crtEYIB* with trc promoter	[22]
Plasmids		
pSC101	Low copy plasmid	Unpublished
pACYC184-M	*cat*; replace *tet* with *lacI* and *Ptrc* of pTrc99A-M	[27]
pACYC184M2-Pm46	*cat*; *kan*; expression vector; replace *lacI* and *Ptrc* of pAYCA184-M with FRT-*Km*-FRT::M1-46	Unpublished
pSC102	Low copy plasmid, *ori* and *repA* from pSC101, M1-46 promoter, *cat* from pACYC184-M2-Pm46	Unpublished
pYL002	*crtW* in pSC102	Unpublished
pYL501	*crtW* and *crtZ* in pSC102	Unpublished
pGlpF-CrtW	*glpF* fused with *crtW* in pSC102	This study
pCrtZ	*crtZ* in pSC102	This study
pGlpF-CrtZ	*glpF* fused with *crtZ* in pSC102	This study
pGlpF-CrtW/GlpF-CrtZ	*glpF* fused with *crtW* and *glpF* fused with *crtZ* in pSC102	This study
pCrtW-CrtZ	*crtW* fused with *crtZ* in pSC102	This study
pGlpF-CrtW-CrtZ	*glpF* fused with *crtW* and *crtZ* in pSC102	This study
pPhCCD1	*PhCCD1* in pACYC184M2-Pm46	This study
pGlpF-PhCCD1	*glpF* fused with *PhCCD1* in pACYC184M2-Pm46	This study
pMBP-PhCCD1	MBP fused with *PhCCD1* in pACYC184M2-Pm46	This study
pSPompA-PhCCD1	The signal peptide sequence of *ompA* fused with *PhCCD1* in pACYC184M2-Pm46	This study

### Plasmid construction

The backbone fragments were amplified from plasmid pACYC184-M [27], pSC101, or their derivatives. The codon-optimized PhCCD1 gene of *Petunia hybrida* was synthesized by Genewiz (Suzhou, China). The gene fragments GG-PhCCD1 and GG-2PhCCD1 were amplified from the plasmid carrying PhCCD1 using the primer pairs phccd1-F/phccd1-R and 2phccd1-F/phccd1-R, respectively. Fragments containing *crtW* and *crtZ* were amplified from pYL501. PCRs were performed using PrimeSTAR^®^ HS DNA Polymerase (Takara, Japan) and carried out in a thermocycler using the following program: 98 °C for 10 s, X °C for 5 s (step 2), 72 °C for Y min (step 3), repeat step 1–3 for 30 cycles, which X means the Tm of primers minus 5, Y equates the length (Kb) of PCR products. The PCR products were subjected to *DpnI* digestion (10 U, 16 h, 37 °C) and gel purification. The primers used in this study are summarized in Additional file 1: Table S1. Part of primers is designed by j5 DNA Assembly Design Software for scarless ligation [28]. All the primers were designed with linker sequences for Type II restriction enzymes for DNA assembly. The oligonucleotide primers were purchased from GENEWIZ (Suzhou, China).

The DNA fragments were assembled using the Golden Gate DNA assembly method [29, 30]. 100 ng of the vector fragment and equimolar amounts of other DNA parts were mixed in a 20 μL Golden Gate reaction with 1 μL *Bsa*I-HF, 1 μL T4 ligase (New England Biolabs, USA) and 1× T4 ligase buffer. The reaction was carried out in a thermocycler using the following program: 37 °C for 5 min, 37 °C for 5 min (step 2), 16 °C for 10 min (step 3), repeat step 2 − 3 for 20 cycles, 16 °C for 20 min, 37 °C for 30 min, 75 °C for 6 min, and 4 °C hold. 1.5 μL of the resulting reaction solution was used to transform 80 μL of competent cells (Table 1) to obtain the assembled plasmids.

### Measurement of β-ionone and astaxanthin concentrations

To obtain the produced β-ionone, culture samples were centrifuged for 5 min at 3186×*g* to separate the dodecane phase as the upper layer, which was filtered through a 0.22 μm organic nylon filter. Quantitation of β-ionone was performed on a 7890B gas chromatography system (Agilent Technologies, USA) coupled with a flame ionization detector, using a 19091J-413 HP-5 capillary column (30 m × 0.32 mm id, 0.25 μm film thickness; J&W Scientific, Agilent Technologies, USA). Injection of the samples was performed in splitless mode at 250 °C. The oven program comprised 80 °C for 1 min, ramp at 10 °C/min to 120 °C, and 3 °C/min to 240 °C. The concentrations of β-ionone were calculated using a calibration curve comprising 66.25–1060 mg/L of an authentic β-ionone standard purchased from Sigma (Sigma-Aldrich, St. Luis, MO, USA).

To obtain the produced astaxanthin (including precursors of zeaxanthin, canthaxanthin, and β-carotene), a 2-mL culture was centrifuged at 16,200×*g* for 3 min to obtain the cell pellet, which was washed with sterile water and centrifuged at 16,200×*g* for 3 min to obtain the cleaned pellet. Subsequently, 750 μL of the extraction solution (acetonitrile/methanol/dichloromethane, 21:21:8, v/v/v) was added to the pellet and ultrasonicated in the ice bath for 30 min, after which the resulting lysate was centrifuged at 16,200×*g* for 3 min. The resulting supernatant was transferred to a fresh centrifuge tube and another 750 μL of extraction solution was added to perform the extraction again. The supernatants of both extractions were combined, centrifuged at 16,200×*g* for 3 min, and the resulting combined supernatant filtered through a 0.22 μm organic nylon filter before astaxanthin content was measured by high-performance liquid chromatography (HPLC). The HPLC was performed on a Series 1200 system (Agilent Technologies, Agilent, USA) with a variable wavelength detector and a Symmetry C18 column (250 mm × 4.6 mm, 5 μm, Waters Ireland). The column was eluted at a rate of 0.8 mL/min with a linear gradient from 80% solvent D (acetonitrile/methanol/dichloromethane, 21:21:8, v/v/v) and 20% solvent B (methanol/water, 1:9, v/v) to 100% solvent D for 18 min, followed by a gradient from 100% solvent D to 80% solvent D and 20% solvent B for 7 min. The final solvent ratio was kept for 10 min to re-equilibrate the column. The content of the carotenoids including astaxanthin, zeaxanthin, canthaxanthin, and β-carotene was determined from the HPLC peak area at 476 nm. Concentrations of carotenoids were calculated using a calibration curve comprising an authentic reference standard purchased from Sigma (Sigma-Aldrich, St. Luis, MO, USA).

## Results and discussion

### Targeting PhCCD1 to different cell compartments for optimal catalytic efficiency

**Fig. 4:**
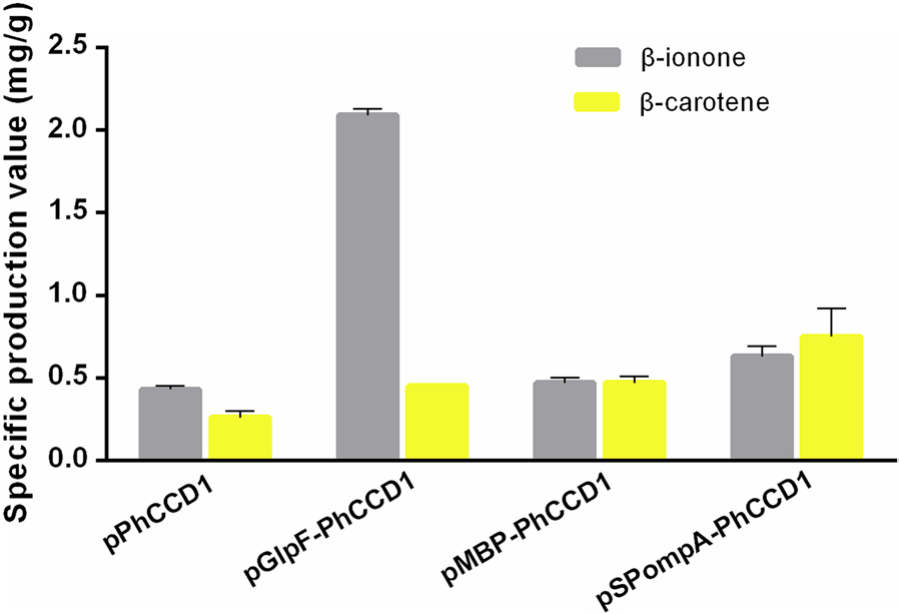
The specific β-ionone and β-carotene production with differently localized PhCCD1

Since PhCCD1 is a cytoplasmic protein and the substrate β-carotene is membrane bound, its position in the heterologous host might not be optimal. In this study, *E. coli* localizing tags were applied to target PhCCD1 to different cell compartments to find the optimal location for its catalytic activity. We fused GlpF [31, 32] to PhCCD1 to position it in the inner membrane, to the *E. coli* maltose-binding protein (MBP, without the signal peptide) to position it in the cytoplasm and increase its solubility [33], and to the signal peptide of OmpA to position it in the periplasmic space [34]. All the proteins or polypeptide were fused to the N-terminal of PhCCD1. Plasmids carrying these fusion proteins were introduced into the β-carotene producing strain CAR003, and the resulting transformants were subjected to shake-flask fermentation. As can be seen in Fig. 4, the position of PhCCD1 had tremendous impact on the production of β-ionone. While the strains with PhCCD1 in the cytoplasm and periplasmic space had similar β-ionone yields as the control with no positioning tags, the strain with the membrane-localized PhCCD1 had tremendously improved production, reaching 386.0% of the control. Since PhCCD1 catalyzes the production of β-ionone from β-carotene in a simple one-step reaction, the results suggest that β-carotene is mainly associated with the membrane compartment, which means that localizing other β-carotene-converting enzymes to the membrane is a feasible strategy to improve their catalytic efficiency. As a corollary, the results also suggest that β-carotene content in either the cytoplasm or the periplasmic space is low, and the efficiency of β-carotene-converting enzymes is, therefore, diminished in these cell compartments. This might be true for most reactions with relatively large hydrophobic substrates.

### Targeting CrtW and CrtZ to the membrane for improved canthaxanthin and zeaxanthin production

**Fig. 5:**
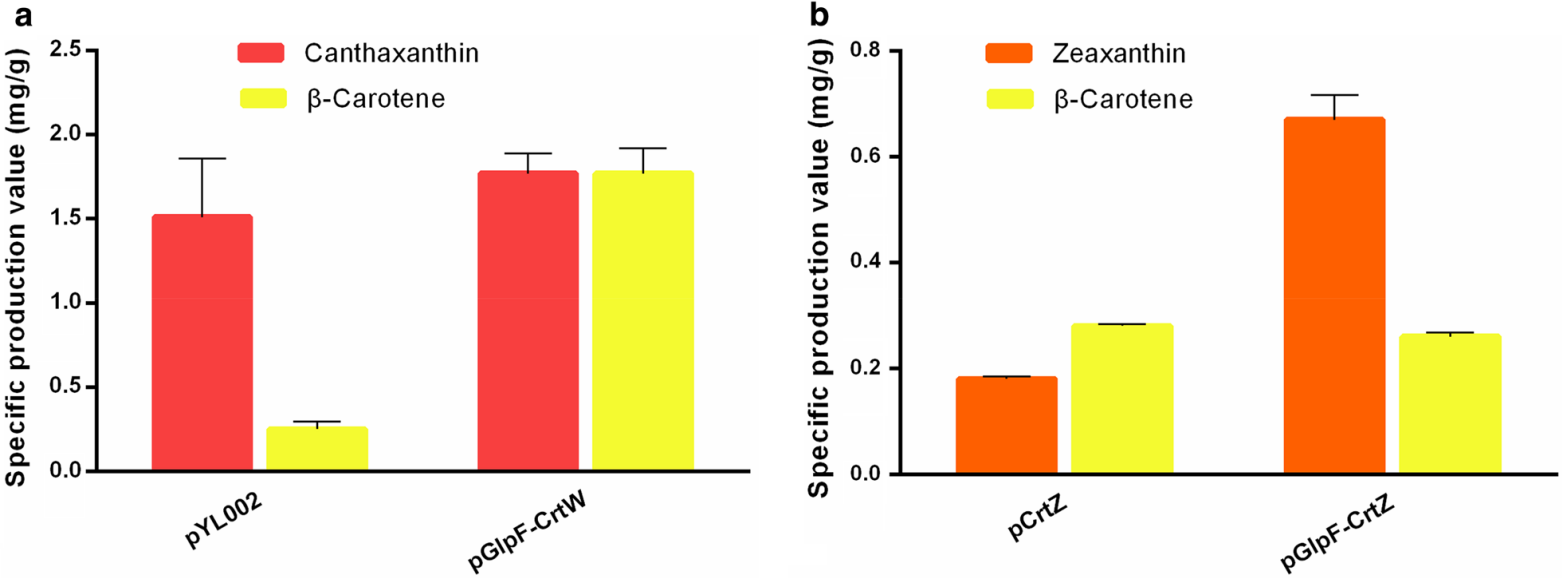
The specific canthaxanthin and zeaxanthin production by membrane-localized CrtW (**a**), and CrtZ (**b**)

As illustrated in Fig. 1, canthaxanthin and zeaxanthin are not only intermediates of astaxanthin, but also high-value natural products themselves [35–38]. Both are derivatives of β-carotene and each is produced by a single enzyme via a two-step reaction. With such reactions, we have proved that targeting the enzymes to the membrane was able to improve their catalytic efficiency. Thus, GlpF was fused to the N-terminal of CrtW and CrtZ, and expressed in the β-carotene-producing *E. coli* CAR003 to produce canthaxanthin and zeaxanthin, respectively (Fig. 5). While the yield of canthaxanthin was only 17.2% higher than that of the control strain with no GlpF fusion (Fig. 5a) which is not a significant increase due to the larger standard deviation of control, the yield of zeaxanthin was significantly increased, reaching 272.2% of the control (Fig. 5b). Nevertheless, localizing CrtW and CrtZ generally increased their catalytic efficiency, similar to PhCCD1. This observation further indicated that the membrane-localization strategy may be universally applicable to β-carotene-converting enzymes, and may be even to other enzymes with large hydrophobic substrates.

### Optimizing the localization of astaxanthin-synthesis enzymes for improved production

The heterologous synthesis pathway of astaxanthin comprises two enzymes, CrtW and CrtZ, which perform four interchangeable reactions initiated from β-carotene (Fig. 1). Although we have demonstrated that localizing β-carotene-converting enzymes to the membrane could improve their catalytic efficiency, optimal localization of the astaxanthin synthesis pathway enzymes remained a more complex problem. As illustrated in Fig. 2c, d, there are at least three localization configurations for the two enzymes CrtW and CrtZ and their membrane-bound substrate. They can be both localized to the membrane separately (Fig. 2c), linked and localized to the membrane (Fig. 2d) or just linked without the membrane-targeting tag (Fig. 3). The localization situation of CrtW and CrtZ, therefore, needed to be discussed in detail to find an optimal configuration for maximizing the production of astaxanthin.

**Fig. 6:**
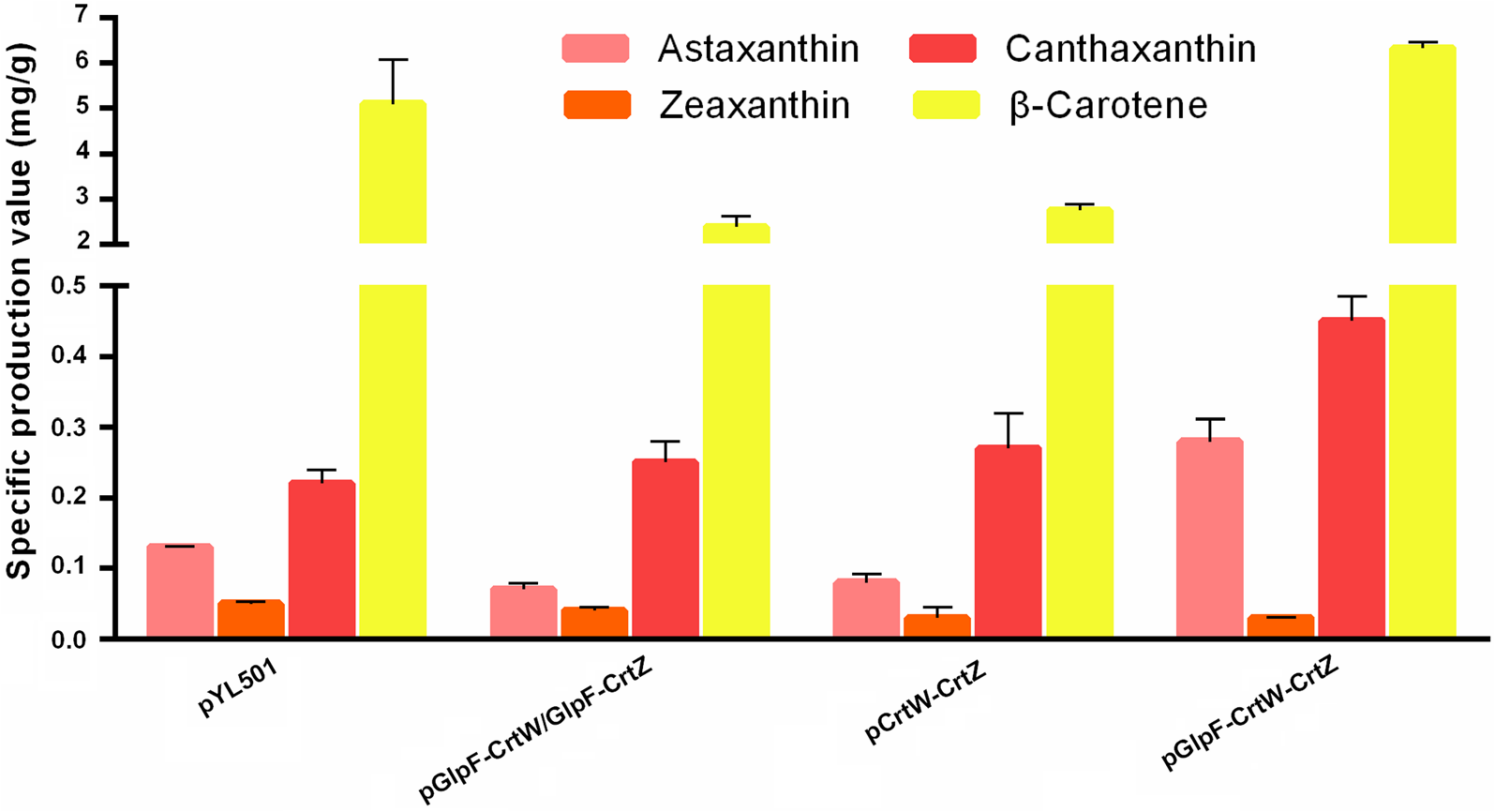
The specific production of astaxanthin, canthaxanthin, zeaxanthin and β-carotene with differently localized CrtW and CrtZ

To localize both CrtW and CrtZ to the membrane (Fig. 2c), GlpF was fused to them individually. To link CrtW and CrtZ together, linker-2, a flexible eight-amino acid linker (Additional file 1: Table S1), was used to fuse them into one protein CrtW–CrtZ (Fig. 3). Furthermore, GlpF was fused to the fusion protein CrtW-CrtZ to position it to the membrane (Fig. 2d). CrtW and CrtZ constructs in these three localization configurations were expressed in the β-carotene-producing strain CAR025 to produce astaxanthin. As illustrated in Fig. 6, individually located CrtW and CrtZ (pGlpF–CrtW/GlpF–CrtZ) and their fusion protein CrtW–CrtZ (pCrtW–CrtZ) actually showed reduced astaxanthin specific production compared to the control strain CAR025 (pYL501), which having CrtW and CrtZ expressed in one operon with their own RBSs. By contrast, the membrane-targeted CrtW–CrtZ (pGlpF–CrtW–CrtZ) had increased catalytic efficiency and achieved a yield 215.4% of that of the control strain. While the zeaxanthin yield of all groups was low, the canthaxanthin accumulation was higher than zeaxanthin, which may be due to the lower enzyme activity of CrtW than CrtZ. In addition, β-carotene accumulation was much higher than the both, which was probably also caused by difference in enzyme activities.

These interesting results demonstrated that different localization strategies actually had tremendous impact on the pathway efficiency. While two of the three configurations we tested decreased the catalytic efficiency, it was serendipitous to obtain one with significantly improved efficiency. In the configuration with separately membrane-bound CrtW and CrtZ, their efficiency might be increased individually. However, since the products of CrtW and CrtZ each serve as the substrates for the other enzyme (Fig. 1), the separately localized CrtW and CrtZ on the membrane might still be distant from their respective substrates for astaxanthin production (Fig. 2c). Furthermore, CrtW and CrtZ were not able to move as freely to bind their substrates as in the control strain, where they are in the cytoplasm and hence may have had decreased efficiency due at least in part to lower mobility. While the fusion protein CrtW–CrtZ should have the advantage of close proximity to each other (Fig. 3), the scarce β-carotene content in the cytoplasm could have diminished such effects. In the optimal configuration with CrtW and CrtZ linked and localized to the membrane together (Fig. 2d), theoretically, the substrates for all reaction steps were brought to the vicinity of the corresponding enzymes (Fig. 1), which resulted in the observed drastically improved catalytic efficiency.

## Conclusion

There were few reports regarding the optimization of synthetic pathways by localization of multiple enzymes, especially when the substrates were membrane bound. In this work, *E. coli* localizing tags were applied to position PhCCD1 into different cell compartments, and the optimal location for its catalytic efficiency was found to be in the membrane, as expected. The results suggested that β-carotene was mainly associated with the membrane compartment, and, hence, targeting β-carotene-converting enzymes to the membrane is a feasible strategy to improve their catalytic efficiency. As a corollary, the results suggest that the β-carotene content in either the cytoplasmic or periplasmic compartments is too low for efficient enzymatic conversion, which might also be true for other large hydrophobic substrates.

How the localization situation of CrtW and CrtZ affects astaxanthin production was exhaustively investigated in this work, and an optimal positioning strategy was found, whereby CrtW and CrtZ were linked with a flexible linker and localized to the membrane via a GlpF protein fusion. Enzymes in the optimal localization configuration allowed a 215.4% increase of astaxanthin production. This work exploits a localization situation involving membrane-bound substrates, intermediates and multiple enzymes for the first time and provides a workable positioning strategy to solve problems in similar circumstances.

## Additional files

**Additional file 1: Table S1.** Oligonucleotides used in this study.

**Additional file 2.** Plasmid maps and DNA sequences.

## Abbreviations

RBS: ribosome-binding site
DCW: dry cell weight
MEP: 2C-methyl-d-erythritol-4-phosphate

## References

[1] Ajikumar PK, Tyo K, Carlsen S, Mucha O, Phon TH, Stephanopoulos G (2008). Terpenoids: opportunities for biosynthesis of natural product drugs using engineered microorganisms. Mol Pharm.

[2] Das A, Yoon SH, Lee SH, Kim JY, Oh DK, Kim SW (2007). An update on microbial carotenoid production: application of recent metabolic engineering tools. Appl Microbiol Biotechnol.

[3] Schmidtdannert C (2000). Engineering novel carotenoids in microorganisms. Curr Opin Biotechnol.

[4] Doshi R, Nguyen T, Chang G (2013). Transporter-mediated biofuel secretion. Proc Natl Acad Sci USA.

[5] Meckel RA, Kester AS (1980). Extractability of carotenoid pigments from nonphotosynthetic bacteria with solvents and detergents: implications for the location and binding of the pigments. Microbiology.

[6] Mathews MM, Sistrom WR (1959). Intracellular location of carotenoid pigments and some respiratory enzymes in *Sarcina lutea*. J Bacteriol.

[7] Farhi M, Marhevka E, Masci T, Marcos E, Eyal Y, Ovadis M, Abeliovich H, Vainstein A (2011). Harnessing yeast subcellular compartments for the production of plant terpenoids. Metab Eng.

[8] Avalos JL, Fink GR, Stephanopoulos G (2013). Compartmentalization of metabolic pathways in yeast mitochondria improves the production of branched-chain alcohols. Nat Biotechnol.

[9] Lv X, Wang F, Zhou P, Ye L, Xie W, Xu H, Yu H (2016). Dual regulation of cytoplasmic and mitochondrial acetyl-CoA utilization for improved isoprene production in *Saccharomyces cerevisiae*. Nat Commun.

[10] Dueber JE, Wu GC, Malmirchegini GR, Moon TS, Petzold CJ, Ullal AV, Prather KL, Keasling JD (2009). Synthetic protein scaffolds provide modular control over metabolic flux. Nat Biotechnol.

[11] Lee MJ, Mantell J, Hodgson L, Alibhai D, Fletcher JM, Brown IR, Frank S, Xue WF, Verkade P, Woolfson DN, Warren MJ (2018). Engineered synthetic scaffolds for organizing proteins within the bacterial cytoplasm. Nat Chem Biol.

[12] Conrado RJ, Wu GC, Boock JT, Xu H, Chen SY, Lebar T, Turnsek J, Tomsic N, Avbelj M, Gaber R (2012). DNA-guided assembly of biosynthetic pathways promotes improved catalytic efficiency. Nucleic Acids Res.

[13] Zhu LY, Qiu XY, Zhu LY, Wu XM, Zhang Y, Zhu QH, Fan DY, Zhu CS, Zhang DY (2016). Spatial organization of heterologous metabolic system in vivo based on TALE. Sci Rep.

[14] Myhrvold C, Polka JK, Silver PA (2016). Synthetic lipid-containing scaffolds enhance production by colocalizing enzymes. ACS Synth Biol.

[15] Choudhary S, Quin MB, Sanders MA, Johnson ET, Schmidt-Dannert C (2012). Engineered protein nano-compartments for targeted enzyme localization. PLoS ONE.

[16] Jakobson CM, Slininger Lee MF, Tullman-Ercek D (2017). De novo design of signal sequences to localize cargo to the 1,2-propanediol utilization microcompartment. Protein Sci.

[17] Rodriguez-Bustamante E, Sanchez S (2007). Microbial production of C13-norisoprenoids and other aroma compounds via carotenoid cleavage. Crit Rev Microbiol.

[18] Lopez J, Essus K, Kim IK, Pereira R, Herzog J, Siewers V, Nielsen J, Agosin E (2015). Production of beta-ionone by combined expression of carotenogenic and plant CCD1 genes in *Saccharomyces cerevisiae*. Microb Cell Fact.

[19] Rubio A, Rambla JL, Santaella M, Gomez MD, Orzaez D, Granell A, Gomez-Gomez L (2008). Cytosolic and plastoglobule-targeted carotenoid dioxygenases from *Crocus sativa* are both involved in beta-ionone release. J Biol Chem.

[20] Frusciante S, Diretto G, Bruno M, Ferrante P, Pietrella M, Prado-Cabrero A, Rubio-Moraga A, Beyer P, Gomez-Gomez L, Al-Babili S (2014). Novel carotenoid cleavage dioxygenase catalyzes the first dedicated step in saffron crocin biosynthesis. Proc Natl Acad Sci USA.

[21] Artimo P, Jonnalagedda M, Arnold K, Baratin D, Csardi G, de Castro E, Duvaud S, Flegel V, Fortier A, Gasteiger E (2012). ExPASy: SIB bioinformatics resource portal. Nucleic Acids Res.

[22] Wu T, Ye L, Zhao D, Li S, Li Q, Zhang B, Bi C, Zhang X (2017). Membrane engineering—a novel strategy to enhance the production and accumulation of beta-carotene in *Escherichia coli*. Metab Eng.

[23] Lee PC, Schmidt-Dannert C (2002). Metabolic engineering towards biotechnological production of carotenoids in microorganisms. Appl Microbiol Biotechnol.

[24] Nishida Y, Adachi K, Kasai H, Shizuri Y, Shindo K, Sawabe A, Komemushi S, Miki W, Misawa N (2005). Elucidation of a carotenoid biosynthesis gene cluster encoding a novel enzyme, 2,2′-beta-hydroxylase, from *Brevundimonas* sp. strain SD212 and combinatorial biosynthesis of new or rare xanthophylls. Appl Environ Microbiol.

[25] Choi SK, Nishida Y, Matsuda S, Adachi K, Kasai H, Peng X, Komemushi S, Miki W, Misawa N (2005). Characterization of beta-carotene ketolases, CrtW, from marine bacteria by complementation analysis in *Escherichia coli*. Mar Biotechnol (NY).

[26] Scaife MA, Burja AM, Wright PC (2009). Characterization of cyanobacterial beta-carotene ketolase and hydroxylase genes in *Escherichia coli*, and their application for astaxanthin biosynthesis. Biotechnol Bioeng.

[27] Zhao J, Li Q, Sun T, Zhu X, Xu H, Tang J, Zhang X, Ma Y (2013). Engineering central metabolic modules of *Escherichia coli* for improving β-carotene production. Metab Eng.

[28] Hillson NJ, Rosengarten RD, Keasling JD (2012). j5 DNA assembly design automation software. ACS Synth Biol.

[29] Engler C, Marillonnet S (2011). Generation of families of construct variants using golden gate shuffling. Methods Mol Biol.

[30] Liang J, Chao R, Abil Z, Bao Z, Zhao H (2014). FairyTALE: a high-throughput TAL effector synthesis platform. ACS Synth Biol.

[31] Fu D, Libson A, Miercke LJW, Weitzman C, Nollert P, Krucinski J, Stroud RM (2000). Structure of a glycerol-conducting channel and the basis for its selectivity. Science.

[32] Neophytou I, Harvey R, Lawrence J, Marsh P, Panaretou B, Barlow D (2007). Eukaryotic integral membrane protein expression utilizing the *Escherichia coli* glycerol-conducting channel protein (GlpF). Appl Microbiol Biotechnol.

[33] Raran-Kurussi S, Keefe K, Waugh DS (2015). Positional effects of fusion partners on the yield and solubility of MBP fusion proteins. Protein Expr Purif.

[34] Yamabhai M, Emrat S, Sukasem S, Pesatcha P, Jaruseranee N, Buranabanyat B (2008). Secretion of recombinant Bacillus hydrolytic enzymes using *Escherichia coli* expression systems. J Biotechnol.

[35] Hulisz DT, Boles GL (1993). Clinical review of canthaxanthin (6Tanning Pills’): before recommending this product for tanning long-term safety must be established. Am Pharm.

[36] Ma L, Yan SF, Huang YM, Lu XR, Qian F, Pang HL, Xu XR, Zou ZY, Dong PC, Xiao X (2012). Effect of lutein and zeaxanthin on macular pigment and visual function in patients with early age-related macular degeneration. Ophthalmology.

[37] Palozza P, Maggiano N, Calviello G, Lanza P, Piccioni E, Ranelletti FO, Bartoli GM (1998). Canthaxanthin induces apoptosis in human cancer cell lines. Carcinogenesis.

[38] Zhao L, Sweet BV (2008). Lutein and zeaxanthin for macular degeneration. Am J Health-Syst Pharm.

