# Supplementary material for: Optimizing the localization of astaxanthin enzymes for improved productivity
Source: Biotechnol Biofuels. 2018 Oct 10;11:278. doi: 10.1186/s13068-018-1270-1 (PMC6180651; doi:10.1186/s13068-018-1270-1)
Supplement: Supplementary file 1 — Additional file 1: Table S1. Oligonucleotides used in this study. [file 13068_2018_1270_MOESM1_ESM.docx]

**Table S1** Oligonucleotides used in this study.

| Primers | Sequence | Source |
| --- | --- | --- |
| Ga9-184-F | CCAGGTCTCACCAGAGCCTGATACAGATTAAATCAGAAC | This study |
| Ga9-184-R | CCAGGTCTCAGAGCAAGCTGTCAAACATGAGAATTAC | This study |
| Ga1-M46-F | CCAGGTCTCAGCTCCGCTTGGATGGCATCCTGCCTTG | This study |
| Ga1-M46-R | CCAGGTCTCAACCGGTTTCCTGGTTTAAACCGAATTG | This study |
| 2GlpF-F | CCACGTCTCACGGTATGAGTCAAACATCAACCTTGAAAG | This study |
| 2GlpF-R | CCACGTCTCAATCAGCGAAGCTTTTTGTTCTGAAGGAGTTGTGG | This study |
| phccd1-F | CCACGTCTCATGATGGGTCGTAAAGAAAGCGATGATG | This study |
| phccd1-R | CCACGTCTCACTGGTTACAGTTTTGCCTGCTCCTGGATCTG | This study |
| 2phccd1-F | CCACGTCTCACGGTATGGGTCGTAAAGAAAGCGATGATG | This study |
| SA-DW-PhCCD1-F | CACCAGGTCTCAGGCACTGGCTGGTTTCGCTACCGTAGCGCAGGCCATGGGTCGTAAAGAAAGCGATGATGGCG | This study |
| SA-UP-PhCCD1-R | CACCAGGTCTCATGCCACTGCAATCGCGATAGCTGTCTTTTTCATACCGGTTTCCTGGTTTAAACCGAATTGGTGG | This study |
| MBP-PC1-F | CACCAGGTCTCAGTATGAAAATCGAAGAAGGTAAACTGGTAATCTGG | This study |
| MBP-PC1-R | CACCAGGTCTCATAGTCTGCGCGTCTTTCAGGGC | This study |
| M46-PC1-F | CACCAGGTCTCAACTATGGGTCGTAAAGAAAGCGATGATGGCG | This study |
| M46-PC1-R | CACCAGGTCTCAATACCGGTTTCCTGGTTTAAACCGAATTGGTGG | This study |
| YL002-F | CCAGGTCTCATGATGACCGCCGCAGTCGCA | This study |
| YL002-R | CCAGGTCTCATAGCTGTTTCCTGGTTTAAACCGAATTGGTGG | This study |
| GlpF-F | CCAGGTCTCAGCTATGAGTCAAACATCAACCTTGAAAGGCCA | This study |
| GlpF-R | CCAGGTCTCAATCAGCGAAGCTTTTTGTTCTGAAGGAGTTGTGG | This study |
| GG-crtZ-F | CACCAGGTCTCATGATGTTGTGGATTTGGAATGCCCTGATTGTTCTGG | This study |
| GG-crtZ-F2 | CACCAGGTCTCAGCTATGTTGTGGATTTGGAATGCCCTGATTGTTCTGG | This study |
| GF-WLZ2-F | CACCAGGTCTCATTTCACGATTCACCACGCCACAGACG | This study |
| GF-WLZ2-R | CACCAGGTCTCATGATGTTGTGGATTTGGAATGCCC | This study |
| GlpF-F | CACCAGGTCTCAATCAGCGAAGCTTTTTGTTCTGAAGGAGTTGTGG | This study |
| GlpF-RBS-R | CACCAGGTCTCAGAAAACCAGGAAACAGCTATGAGTCAAACATCAACCTTGAAAGGCCAGTGC | This study |
| linker-2 | CGAACTTAAGCACCAGGCTGTATT | This study |
| YL002-F2 | CCAGGTCTCAGCTATGACCGCCGCAGTCGCA | This study |
| GG-GFPC1-F3 | CACCAGGTCTCAAACCGGGCACCGAATGTGAAGAGG | This study |
| GG-GFPC1-R | CACCAGGTCTCAATCAGTTTTGCCTGCTCCTGGATCTGC | This study |
| GG-GF002-R | CACCAGGTCTCAGATTCACCACGCCACAGACGCCA | This study |
| GG-SCGFZ-F | CACCAGGTCTCACCAGGGTACCCGGGGATCCTCTAGAGTCG | This study |
| GG-SCGFZ-R | CACCAGGTCTCAATCTTCCCGGGTGGCGCGTC | This study |
