# Supplementary material for: Optimizing the localization of astaxanthin enzymes for improved productivity
Source: Biotechnol Biofuels. 2018 Oct 10;11:278. doi: 10.1186/s13068-018-1270-1 (PMC6180651; doi:10.1186/s13068-018-1270-1)
Supplement: Supplementary file 2 — Additional file 2. Plasmid maps and DNA sequences. [file 13068_2018_1270_MOESM2_ESM.docx]

**Supplementary plasmid profiles and gene sequences**

**pYL501**

LOCUS pYL501 4813 bp DNA circular SYN 19-MAR-1980

DEFINITION Ligation of inverted SacI+RBS+crtZ+KpnI* into pYL002*.

ACCESSION pYL501

SOURCE Unknown.

ORGANISM Unknown

Unclassified.

REFERENCE 1 (bases 1 to 4813)

AUTHORS Self

JOURNAL Unpublished.

COMMENT SECID/File created by SciEd Central, Scientific & Educational Software

COMMENT This file is created by Vector NTI

http://www.invitrogen.com/

COMMENT ORIGDB|GenBank

COMMENT LSOWNER|

COMMENT VNTNAME|pYL501|

FEATURES Location/Qualifiers

rep_origin 130..486

/gene="ori"

/vntifkey="33"

/label=ori

CDS 510..1460

/gene="repA"

/vntifkey="4"

/label=repA

CDS 2081..2740

/gene="Cm"

/vntifkey="4"

/label=Cm

terminator 2992..3292

/gene="rrnB"

/vntifkey="43"

/label=rrnB

CDS complement (3445..3972)

/gene="crtZ"

/vntifkey="4"

/label=crtZ

CDS complement (3991..4725)

/gene="crtW"

/vntifkey="4"

/label=crtW

promoter complement(4742..4813)

/gene="'M1-46"

/vntifkey="30"

/label=M1-46

BASE COUNT 1421 a 1130 c 1122 g 1140 t

ORIGIN

1 gcgcctgtag tgccatttac ccccattcac tgccagagcc gtgagcgcag cgaactgaat

61 gtcacgaaaa agacagcgac tcaggtgcct gatggtcgga gacaaaagga atattcagcg

121 atttgcccga gcttgcgagg gtgctactta agcctttagg gttttaaggt ctgttttgta

181 gaggagcaaa cagcgtttgc gacatccttt tgtaatactg cggaactgac taaagtagtg

241 agttatacac agggctggga tctattcttt ttatcttttt ttattctttc tttattctat

301 aaattataac cacttgaata taaacaaaaa aaacacacaa aggtctagcg gaatttacag

361 agggtctagc agaatttaca agttttccag caaaggtcta gcagaattta cagataccca

421 caactcaaag gaaaaggact agtaattatc attgactagc ccatctcaat tggtatagtg

481 attaaaatca cctagaccaa ttgagatgta tgtctgaatt agttgttttc aaagcaaatg

541 aactagcgat tagtcgctat gacttaacgg agcatgaaac caagctaatt ttatgctgtg

601 tggcactact caaccccacg attgaaaacc ctacaaggaa agaacggacg gtatcgttca

661 cttataacca atacgctcag atgatgaaca tcagtaggga aaatgcttat ggtgtattag

721 ctaaagcaac cagagagctg atgacgagaa ctgtggaaat caggaatcct ttggttaaag

781 gctttgagat tttccagtgg acaaactatg ccaagttctc aagcgaaaaa ttagaattag

841 tttttagtga agagatattg ccttatcttt tccagttaaa aaaattcata aaatataatc

901 tggaacatgt taagtctttt gaaaacaaat actctatgag gatttatgag tggttattaa

961 aagaactaac acaaaagaaa actcacaagg caaatataga gattagcctt gatgaattta

1021 agttcatgtt aatgcttgaa aataactacc atgagtttaa aaggcttaac caatgggttt

1081 tgaaaccaat aagtaaagat ttaaacactt acagcaatat gaaattggtg gttgataagc

1141 gaggccgccc gactgatacg ttgattttcc aagttgaact agatagacaa atggatctcg

1201 taaccgaact tgagaacaac cagataaaaa tgaatggtga caaaatacca acaaccatta

1261 catcagattc ctacctacgt aacggactaa gaaaaacact acacgatgct ttaactgcaa

1321 aaattcagct caccagtttt gaggcaaaat ttttgagtga catgcaaagt aagcatgatc

1381 tcaatggttc gttctcatgg ctcacgcaaa aacaacgaac cacactagag aacatactgg

1441 ctaaatacgg aaggatctga ggttcttatg gctcttgtat ctatcagtga agcatcaaga

1501 ctaacaaaca aaagtagaac aactgttcac cgttagatat caaagggaaa actgtccata

1561 tgcacagatg aaaacggtgt aaaaaagata gatacatcag agcttttacg agtttttggt

1621 gcatttaaag ctgttcacca tgaacagatc gacaatgtaa cagatgaaca gcatgtaaca

1681 cctaatagaa caggtgaaac cagtaaaaca aagcaactag aacatgaaat tgaacacctg

1741 agacaacttg ttacagctca acagtcacac atagacagcc tgaaacaggc gatgctgctt

1801 atcgaatcaa agctgccgac aacacgggag ccagtgacgc ctcccgtggg gaaaaaatca

1861 tggcaattct ggaagaaata gcgctgtgac ggaagatcac ttcgcagaat aaataaatcc

1921 tggtgtccct gttgataccg ggaagccctg ggccaacttt tggcgaaaat gagacgttga

1981 tcggcacgta agaggttcca actttcacca taatgaaata agatcactac cgggcgtatt

2041 ttttgagtta tcgagatttt caggagctaa ggaagctaaa atggagaaaa aaatcactgg

2101 atataccacc gttgatatat cccaatggca tcgtaaagaa cattttgagg catttcagtc

2161 agttgctcaa tgtacctata accagaccgt tcagctggat attacggcct ttttaaagac

2221 cgtaaagaaa aataagcaca agttttatcc ggcctttatt cacattcttg cccgcctgat

2281 gaatgctcat ccggaattcc gtatggcaat gaaagacggt gagctggtga tatgggatag

2341 tgttcaccct tgttacaccg ttttccatga gcaaactgaa acgttttcat cgctctggag

2401 tgaataccac gacgatttcc ggcagtttct acacatatat tcgcaagatg tggcgtgtta

2461 cggtgaaaac ctggcctatt tccctaaagg gtttattgag aatatgtttt tcgtctcagc

2521 caatccctgg gtgagtttca ccagttttga tttaaacgtg gccaatatgg acaacttctt

2581 cgcccccgtt ttcaccatgg gcaaatatta tacgcaaggc gacaaggtgc tgatgccgct

2641 ggcgattcag gttcatcatg ccgtctgtga tggcttccat gtcggcagaa tgcttaatga

2701 attacaacag tactgcgatg agtggcaggg cggggcgtaa tttttttaag gcagttattg

2761 gtgcccttaa acgcctggtg ctacgcctga ataagtgata ataagcggat gaatggcaga

2821 aattcgaaag caaattcgac ccggtcgtcg gttcagggca gggtcgttaa atagccgctt

2881 atgtctattg ctggtttacc ggtttattga ctaccggaag cagtgtgacc gtgtgcttct

2941 caaatgcctg aggccagttt gctcaggctc tcccatttaa atagaaacgc aaaaaggcca

3001 tccgtcagga tggccttctg cttaatttga tgcctggcag tttatggcgg gcgtcctgcc

3061 cgccaccctc cgggccgttg cttcgcaacg ttcaaatccg ctcccggcgg atttgtccta

3121 ctcaggagag cgttcaccga caaacaacag ataaaacgaa aggcccagtc tttcgactga

3181 gcctttcgtt ttatttgatg cctggcagtt ccctactctc gcatggggag accccacact

3241 accatcggcg ctacggcgtt tcacttctga gttcggcatg gggtcaggtg ggaccaccgc

3301 gctactgccg ccaggcaaat tctgttttat cagaccgctt ctgcgttctg atttaatctg

3361 tatcaggctg aaaatcttct ctcatccgcc aaaacagcca agcttgcatg cctgcaggtc

3421 gactctagag gatccccggg taccttactt cccgggtggc gcgtcacgct ccacgctcag

3481 ctgatctctg gcagcgcccc gtttaacgcc atgacgttct cgcagcgtcg cctgcagttt

3541 tgacagcggc ggcgcgtaaa gaaagccaaa cgagacacag ccctctttgc ctcgtaccgc

3601 atgatgcatg cggtgcgcca tgtaaagcct gcgtaaataa ccccggcgcg gaacatagcg

3661 gaatggccag cgctgatgta ccaggccatc atgcacaata aaatagagca ggccgtagag

3721 cgtcataccc gcgccgatcc actgcaaggg ccagacaccc gtgctgccca gataaatgag

3781 caaaatcgac agagcggcga acactacggc atagaggtca ttaacctcaa accagccttt

3841 atgcggttca tgatgtgaca gatgccagcc ccaaccccag ccatgcataa tgtatctgtg

3901 cgccagtgca gccgttatct ccattccgat aacagtgacc agaacaatca gggcattcca

3961 aatccacaac atagctgttt cctggagctc ttacgattca ccacgccaca gacgccacca

4021 cggacgccac ggggtcagat gatgttcatg gtgacgaccc agatggaagc acgtcagcag

4081 agacagaacc ggaccgtaac cggaactacg tgcgtgatgt gcgtctgcaa acggttgatc

4141 ggtgtgacga tgcggcagcc acgtgccgaa ggtaaacatc tgcagtgcgc tcagcagtgc

4201 cggtgctgcc cagaaggtca gcagatttgc cggacgtgca cccagaccaa acagtgcgat

4261 cagaaccaga gccgtcagga ctgccatttc gcgccagccg aagtaggtac ggaaaaagtt

4321 caggaaccac ggcagaaaag cacgcggagc cggtgcataa aagtccgggt catctgccgt

4381 acccggagct gcgtgatgag cgtgatgtgc ggttttcagg cgatcgaaac gaaaaccggc

4441 atacaggccc agggtcaggc ggcccacggc cgcgttcaga cgcggacgac ccggcgccag

4501 cgaaccgtgc atggcatcat gcgcaacaat aaacagaccc actgacagcc acgtctgcac

4561 agcgacgatt gccggaacaa tcaccagaga gctggtgccc caacgatgaa agtagacacc

4621 ataaacatgc agggagcccc agccagcaac aatcatacca gccagggtca ggccaatcca

4681 ggtttgacgc gggacaatac gcggttctgc gactgcggcg gtcatagctg tttcctggtt

4741 taaaccgaat tggtggggcg agaggctcaa ttatatcaac gttgttatct cttgtcaaca

4801 ccgccagaga taa

//

**pYL002**

LOCUS pYL002 4270 bp DNA circular 19-MAR-1980

ACCESSION pYL002

VERSION pYL002

SOURCE

ORGANISM

COMMENT This file is created by Vector NTI

http://www.invitrogen.com/

COMMENT ORIGDB|GenBank

COMMENT LSOWNER|

COMMENT VNTNAME|pYL002|

FEATURES Location/Qualifiers

rep_origin 130..486

/vntifkey="33"

/label=ori

CDS 510..1460

/vntifkey="4"

/label=repA

CDS 2081..2740

/vntifkey="4"

/label=Cm

terminator 2992..3292

/vntifkey="43"

/label=rrnB

CDS complement(3451..4182)

/vntifkey="4"

/label=crtW

promoter complement(4199..4270)

/vntifkey="30"

/label=M1-46

BASE COUNT 1281 a 963 c 990 g 1036 t

ORIGIN

1 gcgcctgtag tgccatttac ccccattcac tgccagagcc gtgagcgcag cgaactgaat

61 gtcacgaaaa agacagcgac tcaggtgcct gatggtcgga gacaaaagga atattcagcg

121 atttgcccga gcttgcgagg gtgctactta agcctttagg gttttaaggt ctgttttgta

181 gaggagcaaa cagcgtttgc gacatccttt tgtaatactg cggaactgac taaagtagtg

241 agttatacac agggctggga tctattcttt ttatcttttt ttattctttc tttattctat

301 aaattataac cacttgaata taaacaaaaa aaacacacaa aggtctagcg gaatttacag

361 agggtctagc agaatttaca agttttccag caaaggtcta gcagaattta cagataccca

421 caactcaaag gaaaaggact agtaattatc attgactagc ccatctcaat tggtatagtg

481 attaaaatca cctagaccaa ttgagatgta tgtctgaatt agttgttttc aaagcaaatg

541 aactagcgat tagtcgctat gacttaacgg agcatgaaac caagctaatt ttatgctgtg

601 tggcactact caaccccacg attgaaaacc ctacaaggaa agaacggacg gtatcgttca

661 cttataacca atacgctcag atgatgaaca tcagtaggga aaatgcttat ggtgtattag

721 ctaaagcaac cagagagctg atgacgagaa ctgtggaaat caggaatcct ttggttaaag

781 gctttgagat tttccagtgg acaaactatg ccaagttctc aagcgaaaaa ttagaattag

841 tttttagtga agagatattg ccttatcttt tccagttaaa aaaattcata aaatataatc

901 tggaacatgt taagtctttt gaaaacaaat actctatgag gatttatgag tggttattaa

961 aagaactaac acaaaagaaa actcacaagg caaatataga gattagcctt gatgaattta

1021 agttcatgtt aatgcttgaa aataactacc atgagtttaa aaggcttaac caatgggttt

1081 tgaaaccaat aagtaaagat ttaaacactt acagcaatat gaaattggtg gttgataagc

1141 gaggccgccc gactgatacg ttgattttcc aagttgaact agatagacaa atggatctcg

1201 taaccgaact tgagaacaac cagataaaaa tgaatggtga caaaatacca acaaccatta

1261 catcagattc ctacctacgt aacggactaa gaaaaacact acacgatgct ttaactgcaa

1321 aaattcagct caccagtttt gaggcaaaat ttttgagtga catgcaaagt aagcatgatc

1381 tcaatggttc gttctcatgg ctcacgcaaa aacaacgaac cacactagag aacatactgg

1441 ctaaatacgg aaggatctga ggttcttatg gctcttgtat ctatcagtga agcatcaaga

1501 ctaacaaaca aaagtagaac aactgttcac cgttagatat caaagggaaa actgtccata

1561 tgcacagatg aaaacggtgt aaaaaagata gatacatcag agcttttacg agtttttggt

1621 gcatttaaag ctgttcacca tgaacagatc gacaatgtaa cagatgaaca gcatgtaaca

1681 cctaatagaa caggtgaaac cagtaaaaca aagcaactag aacatgaaat tgaacacctg

1741 agacaacttg ttacagctca acagtcacac atagacagcc tgaaacaggc gatgctgctt

1801 atcgaatcaa agctgccgac aacacgggag ccagtgacgc ctcccgtggg gaaaaaatca

1861 tggcaattct ggaagaaata gcgctgtgac ggaagatcac ttcgcagaat aaataaatcc

1921 tggtgtccct gttgataccg ggaagccctg ggccaacttt tggcgaaaat gagacgttga

1981 tcggcacgta agaggttcca actttcacca taatgaaata agatcactac cgggcgtatt

2041 ttttgagtta tcgagatttt caggagctaa ggaagctaaa atggagaaaa aaatcactgg

2101 atataccacc gttgatatat cccaatggca tcgtaaagaa cattttgagg catttcagtc

2161 agttgctcaa tgtacctata accagaccgt tcagctggat attacggcct ttttaaagac

2221 cgtaaagaaa aataagcaca agttttatcc ggcctttatt cacattcttg cccgcctgat

2281 gaatgctcat ccggaattcc gtatggcaat gaaagacggt gagctggtga tatgggatag

2341 tgttcaccct tgttacaccg ttttccatga gcaaactgaa acgttttcat cgctctggag

2401 tgaataccac gacgatttcc ggcagtttct acacatatat tcgcaagatg tggcgtgtta

2461 cggtgaaaac ctggcctatt tccctaaagg gtttattgag aatatgtttt tcgtctcagc

2521 caatccctgg gtgagtttca ccagttttga tttaaacgtg gccaatatgg acaacttctt

2581 cgcccccgtt ttcaccatgg gcaaatatta tacgcaaggc gacaaggtgc tgatgccgct

2641 ggcgattcag gttcatcatg ccgtctgtga tggcttccat gtcggcagaa tgcttaatga

2701 attacaacag tactgcgatg agtggcaggg cggggcgtaa tttttttaag gcagttattg

2761 gtgcccttaa acgcctggtg ctacgcctga ataagtgata ataagcggat gaatggcaga

2821 aattcgaaag caaattcgac ccggtcgtcg gttcagggca gggtcgttaa atagccgctt

2881 atgtctattg ctggtttacc ggtttattga ctaccggaag cagtgtgacc gtgtgcttct

2941 caaatgcctg aggccagttt gctcaggctc tcccatttaa atagaaacgc aaaaaggcca

3001 tccgtcagga tggccttctg cttaatttga tgcctggcag tttatggcgg gcgtcctgcc

3061 cgccaccctc cgggccgttg cttcgcaacg ttcaaatccg ctcccggcgg atttgtccta

3121 ctcaggagag cgttcaccga caaacaacag ataaaacgaa aggcccagtc tttcgactga

3181 gcctttcgtt ttatttgatg cctggcagtt ccctactctc gcatggggag accccacact

3241 accatcggcg ctacggcgtt tcacttctga gttcggcatg gggtcaggtg ggaccaccgc

3301 gctactgccg ccaggcaaat tctgttttat cagaccgctt ctgcgttctg atttaatctg

3361 tatcaggctg aaaatcttct ctcatccgcc aaaacagcca agcttgcatg cctgcaggtc

3421 gactctagag gatccccggg taccgagctc cgattcacca cgccacagac gccaccacgg

3481 acgccacggg gtcagatgat gttcatggtg acgacccaga tggaagcacg tcagcagaga

3541 cagaaccgga ccgtaaccgg aactacgtgc gtgatgtgcg tctgcaaacg gttgatcggt

3601 gtgacgatgc ggcagccacg tgccgaaggt aaacatctgc agtgcgctca gcagtgccgg

3661 tgctgcccag aaggtcagca gatttgccgg acgtgcaccc agaccaaaca gtgcgatcag

3721 aaccagagcc gtcaggactg ccatttcgcg ccagccgaag taggtacgga aaaagttcag

3781 gaaccacggc agaaaagcac gcggagccgg tgcataaaag tccgggtcat ctgccgtacc

3841 cggagctgcg tgatgagcgt gatgtgcggt tttcaggcga tcgaaacgaa aaccggcata

3901 caggcccagg gtcaggcggc ccacggccgc gttcagacgc ggacgacccg gcgccagcga

3961 accgtgcatg gcatcatgcg caacaataaa cagacccact gacagccacg tctgcacagc

4021 gacgattgcc ggaacaatca ccagagagct ggtgccccaa cgatgaaagt agacaccata

4081 aacatgcagg gagccccagc cagcaacaat cataccagcc agggtcaggc caatccaggt

4141 ttgacgcggg acaatacgcg gttctgcgac tgcggcggtc atagctgttt cctggtttaa

4201 accgaattgg tggggcgaga ggctcaatta tatcaacgtt gttatctctt gtcaacaccg

4261 ccagagataa

//

**pGlpF-CrtW**

LOCUS pGlpF-CrtW 5116 bp DNA circular SYN 19-MAR-1980

ACCESSION pGlpF-CrtW

SOURCE Unknown.

ORGANISM Unknown

Unclassified.

REFERENCE 1 (bases 1 to 5116)

AUTHORS Self

JOURNAL Unpublished.

COMMENT SECID/File created by SciEd Central, Scientific & Educational Software

COMMENT This file is created by Vector NTI

http://www.invitrogen.com/

COMMENT ORIGDB|GenBank

COMMENT LSOWNER|

COMMENT VNTNAME|pGlpF-CrtW|

FEATURES Location/Qualifiers

terminator complement (762..1062)

/gene="rrnB"

/vntifkey="43"

/label=rrnB

CDS complement (1314..1973)

/gene="Cm"

/vntifkey="4"

/label=Cm

CDS complement (2594..3544)

/gene="repA"

/vntifkey="4"

/label=repA

rep_origin complement (3568..3924)

/gene="ori"

/vntifkey="33"

/label=ori

promoter 4054..4125

/gene="M1-46"

/vntifkey="30"

/label=M1-46

CDS 4142..4984

/gene="GlpF"

/vntifkey="4"

/label=GlpF

CDS 4985..603

/gene="crtW"

/vntifkey="4"

/label=crtW

BASE COUNT 1193 a 1195 c 1189 g 1539 t

ORIGIN

1 agctctctgg tgattgttcc ggcaatcgtc gctgtgcaga cgtggctgtc agtgggtctg

61 tttattgttg cgcatgatgc catgcacggt tcgctggcgc cgggtcgtcc gcgtctgaac

121 gcggccgtgg gccgcctgac cctgggcctg tatgccggtt ttcgtttcga tcgcctgaaa

181 accgcacatc acgctcatca cgcagctccg ggtacggcag atgacccgga cttttatgca

241 ccggctccgc gtgcttttct gccgtggttc ctgaactttt tccgtaccta cttcggctgg

301 cgcgaaatgg cagtcctgac ggctctggtt ctgatcgcac tgtttggtct gggtgcacgt

361 ccggcaaatc tgctgacctt ctgggcagca ccggcactgc tgagcgcact gcagatgttt

421 accttcggca cgtggctgcc gcatcgtcac accgatcaac cgtttgcaga cgcacatcac

481 gcacgtagtt ccggttacgg tccggttctg tctctgctga cgtgcttcca tctgggtcgt

541 caccatgaac atcatctgac cccgtggcgt ccgtggtggc gtctgtggcg tggtgaatcg

601 taagagctcg gtacccgggg atcctctaga gtcgacctgc aggcatgcaa gcttggctgt

661 tttggcggat gagagaagat tttcagcctg atacagatta aatcagaacg cagaagcggt

721 ctgataaaac agaatttgcc tggcggcagt agcgcggtgg tcccacctga ccccatgccg

781 aactcagaag tgaaacgccg tagcgccgat ggtagtgtgg ggtctcccca tgcgagagta

841 gggaactgcc aggcatcaaa taaaacgaaa ggctcagtcg aaagactggg cctttcgttt

901 tatctgttgt ttgtcggtga acgctctcct gagtaggaca aatccgccgg gagcggattt

961 gaacgttgcg aagcaacggc ccggagggtg gcgggcagga cgcccgccat aaactgccag

1021 gcatcaaatt aagcagaagg ccatcctgac ggatggcctt tttgcgtttc tatttaaatg

1081 ggagagcctg agcaaactgg cctcaggcat ttgagaagca cacggtcaca ctgcttccgg

1141 tagtcaataa accggtaaac cagcaataga cataagcggc tatttaacga ccctgccctg

1201 aaccgacgac cgggtcgaat ttgctttcga atttctgcca ttcatccgct tattatcact

1261 tattcaggcg tagcaccagg cgtttaaggg caccaataac tgccttaaaa aaattacgcc

1321 ccgccctgcc actcatcgca gtactgttgt aattcattaa gcattctgcc gacatggaag

1381 ccatcacaga cggcatgatg aacctgaatc gccagcggca tcagcacctt gtcgccttgc

1441 gtataatatt tgcccatggt gaaaacgggg gcgaagaagt tgtccatatt ggccacgttt

1501 aaatcaaaac tggtgaaact cacccaggga ttggctgaga cgaaaaacat attctcaata

1561 aaccctttag ggaaataggc caggttttca ccgtaacacg ccacatcttg cgaatatatg

1621 tgtagaaact gccggaaatc gtcgtggtat tcactccaga gcgatgaaaa cgtttcagtt

1681 tgctcatgga aaacggtgta acaagggtga acactatccc atatcaccag ctcaccgtct

1741 ttcattgcca tacggaattc cggatgagca ttcatcaggc gggcaagaat gtgaataaag

1801 gccggataaa acttgtgctt atttttcttt acggtcttta aaaaggccgt aatatccagc

1861 tgaacggtct ggttataggt acattgagca actgactgaa atgcctcaaa atgttcttta

1921 cgatgccatt gggatatatc aacggtggta tatccagtga tttttttctc cattttagct

1981 tccttagctc ctgaaaatct cgataactca aaaaatacgc ccggtagtga tcttatttca

2041 ttatggtgaa agttggaacc tcttacgtgc cgatcaacgt ctcattttcg ccaaaagttg

2101 gcccagggct tcccggtatc aacagggaca ccaggattta tttattctgc gaagtgatct

2161 tccgtcacag cgctatttct tccagaattg ccatgatttt ttccccacgg gaggcgtcac

2221 tggctcccgt gttgtcggca gctttgattc gataagcagc atcgcctgtt tcaggctgtc

2281 tatgtgtgac tgttgagctg taacaagttg tctcaggtgt tcaatttcat gttctagttg

2341 ctttgtttta ctggtttcac ctgttctatt aggtgttaca tgctgttcat ctgttacatt

2401 gtcgatctgt tcatggtgaa cagctttaaa tgcaccaaaa actcgtaaaa gctctgatgt

2461 atctatcttt tttacaccgt tttcatctgt gcatatggac agttttccct ttgatatcta

2521 acggtgaaca gttgttctac ttttgtttgt tagtcttgat gcttcactga tagatacaag

2581 agccataaga acctcagatc cttccgtatt tagccagtat gttctctagt gtggttcgtt

2641 gtttttgcgt gagccatgag aacgaaccat tgagatcatg cttactttgc atgtcactca

2701 aaaattttgc ctcaaaactg gtgagctgaa tttttgcagt taaagcatcg tgtagtgttt

2761 ttcttagtcc gttacgtagg taggaatctg atgtaatggt tgttggtatt ttgtcaccat

2821 tcatttttat ctggttgttc tcaagttcgg ttacgagatc catttgtcta tctagttcaa

2881 cttggaaaat caacgtatca gtcgggcggc ctcgcttatc aaccaccaat ttcatattgc

2941 tgtaagtgtt taaatcttta cttattggtt tcaaaaccca ttggttaagc cttttaaact

3001 catggtagtt attttcaagc attaacatga acttaaattc atcaaggcta atctctatat

3061 ttgccttgtg agttttcttt tgtgttagtt cttttaataa ccactcataa atcctcatag

3121 agtatttgtt ttcaaaagac ttaacatgtt ccagattata ttttatgaat ttttttaact

3181 ggaaaagata aggcaatatc tcttcactaa aaactaattc taatttttcg cttgagaact

3241 tggcatagtt tgtccactgg aaaatctcaa agcctttaac caaaggattc ctgatttcca

3301 cagttctcgt catcagctct ctggttgctt tagctaatac accataagca ttttccctac

3361 tgatgttcat catctgagcg tattggttat aagtgaacga taccgtccgt tctttccttg

3421 tagggttttc aatcgtgggg ttgagtagtg ccacacagca taaaattagc ttggtttcat

3481 gctccgttaa gtcatagcga ctaatcgcta gttcatttgc tttgaaaaca actaattcag

3541 acatacatct caattggtct aggtgatttt aatcactata ccaattgaga tgggctagtc

3601 aatgataatt actagtcctt ttcctttgag ttgtgggtat ctgtaaattc tgctagacct

3661 ttgctggaaa acttgtaaat tctgctagac cctctgtaaa ttccgctaga cctttgtgtg

3721 ttttttttgt ttatattcaa gtggttataa tttatagaat aaagaaagaa taaaaaaaga

3781 taaaaagaat agatcccagc cctgtgtata actcactact ttagtcagtt ccgcagtatt

3841 acaaaaggat gtcgcaaacg ctgtttgctc ctctacaaaa cagaccttaa aaccctaaag

3901 gcttaagtag caccctcgca agctcgggca aatcgctgaa tattcctttt gtctccgacc

3961 atcaggcacc tgagtcgctg tctttttcgt gacattcagt tcgctgcgct cacggctctg

4021 gcagtgaatg ggggtaaatg gcactacagg cgcttatctc tggcggtgtt gacaagagat

4081 aacaacgttg atataattga gcctctcgcc ccaccaattc ggtttaaacc aggaaacagc

4141 tatgagtcaa acatcaacct tgaaaggcca gtgcattgct gaattcctcg gtaccgggtt

4201 gttgattttc ttcggtgtgg gttgcgttgc agcactaaaa gtcgctggtg cgtcttttgg

4261 tcagtgggaa atcagtgtca tttggggact gggggtggca atggccatct acctgaccgc

4321 aggggtttcc ggcgcgcatc ttaatcccgc tgttaccatt gcattgtggc tgtttgcctg

4381 tttcgacaag cgcaaagtta ttccttttat cgtttcacaa gttgccggcg ctttctgtgc

4441 tgcggcttta gtttacgggc tttactacaa tttatttttc gacttcgagc agactcatca

4501 cattgttcgc ggcagcgttg aaagtgttga tctggctggc actttctcta cttaccctaa

4561 tcctcatatc aattttgtgc aggctttcgc agttgagatg gtgattaccg ctattctgat

4621 ggggctgatc ctggcgttaa cggacgatgg caacggtgta ccacgcggcc ctttggctcc

4681 cttgctgatt ggtctactga ttgcggtcat tggcgcatct atgggcccat tgacaggttt

4741 tgccatgaac ccagcgcgtg acttcggtcc gaaagtcttt gcctggctgg cgggctgggg

4801 caatgtcgcc tttaccggcg gcagagacat tccttacttc ctggtgccgc ttttcggccc

4861 tatcgttggc gcgattgtag gtgcatttgc ctaccgcaaa ctgattggtc gccatttgcc

4921 ttgcgatatc tgtgttgtgg aagaaaagga aaccacaact ccttcagaac aaaaagcttc

4981 gctgatgacc gccgcagtcg cagaaccgcg tattgtcccg cgtcaaacct ggattggcct

5041 gaccctggct ggtatgattg ttgctggctg gggctccctg catgtttatg gtgtctactt

5101 tcatcgttgg ggcacc

//

**pCrtZ**

LOCUS pCrtZ 4060 bp DNA circular SYN 19-MAR-1980

ACCESSION pCrtZ

SOURCE Unknown.

ORGANISM Unknown

Unclassified.

REFERENCE 1 (bases 1 to 4060)

AUTHORS Self

JOURNAL Unpublished.

COMMENT SECID/File created by SciEd Central, Scientific & Educational Software

COMMENT This file is created by Vector NTI

http://www.invitrogen.com/

COMMENT ORIGDB|GenBank

COMMENT LSOWNER|

COMMENT VNTNAME|pCrtZ|

FEATURES Location/Qualifiers

promoter complement (17..88)

/gene="M1-46"

/vntifkey="30"

/label=M1-46

rep_origin 218..574

/gene="ori"

/vntifkey="33"

/label=ori

CDS 598..1548

/gene="repA"

/vntifkey="4"

/label=repA

CDS 2169..2828

/gene="Cm"

/vntifkey="4"

/label=Cm

CDS complement (3533..4060)

/gene="crtZ"

/vntifkey="4"

/label=crtZ

terminator 3080..3380

/vntifkey="43"

/label=rrnB

BASE COUNT 1224 a 909 c 898 g 1029 t

ORIGIN

1 agctgtttcc tggtttaaac cgaattggtg gggcgagagg ctcaattata tcaacgttgt

61 tatctcttgt caacaccgcc agagataagc gcctgtagtg ccatttaccc ccattcactg

121 ccagagccgt gagcgcagcg aactgaatgt cacgaaaaag acagcgactc aggtgcctga

181 tggtcggaga caaaaggaat attcagcgat ttgcccgagc ttgcgagggt gctacttaag

241 cctttagggt tttaaggtct gttttgtaga ggagcaaaca gcgtttgcga catccttttg

301 taatactgcg gaactgacta aagtagtgag ttatacacag ggctgggatc tattcttttt

361 atcttttttt attctttctt tattctataa attataacca cttgaatata aacaaaaaaa

421 acacacaaag gtctagcgga atttacagag ggtctagcag aatttacaag ttttccagca

481 aaggtctagc agaatttaca gatacccaca actcaaagga aaaggactag taattatcat

541 tgactagccc atctcaattg gtatagtgat taaaatcacc tagaccaatt gagatgtatg

601 tctgaattag ttgttttcaa agcaaatgaa ctagcgatta gtcgctatga cttaacggag

661 catgaaacca agctaatttt atgctgtgtg gcactactca accccacgat tgaaaaccct

721 acaaggaaag aacggacggt atcgttcact tataaccaat acgctcagat gatgaacatc

781 agtagggaaa atgcttatgg tgtattagct aaagcaacca gagagctgat gacgagaact

841 gtggaaatca ggaatccttt ggttaaaggc tttgagattt tccagtggac aaactatgcc

901 aagttctcaa gcgaaaaatt agaattagtt tttagtgaag agatattgcc ttatcttttc

961 cagttaaaaa aattcataaa atataatctg gaacatgtta agtcttttga aaacaaatac

1021 tctatgagga tttatgagtg gttattaaaa gaactaacac aaaagaaaac tcacaaggca

1081 aatatagaga ttagccttga tgaatttaag ttcatgttaa tgcttgaaaa taactaccat

1141 gagtttaaaa ggcttaacca atgggttttg aaaccaataa gtaaagattt aaacacttac

1201 agcaatatga aattggtggt tgataagcga ggccgcccga ctgatacgtt gattttccaa

1261 gttgaactag atagacaaat ggatctcgta accgaacttg agaacaacca gataaaaatg

1321 aatggtgaca aaataccaac aaccattaca tcagattcct acctacgtaa cggactaaga

1381 aaaacactac acgatgcttt aactgcaaaa attcagctca ccagttttga ggcaaaattt

1441 ttgagtgaca tgcaaagtaa gcatgatctc aatggttcgt tctcatggct cacgcaaaaa

1501 caacgaacca cactagagaa catactggct aaatacggaa ggatctgagg ttcttatggc

1561 tcttgtatct atcagtgaag catcaagact aacaaacaaa agtagaacaa ctgttcaccg

1621 ttagatatca aagggaaaac tgtccatatg cacagatgaa aacggtgtaa aaaagataga

1681 tacatcagag cttttacgag tttttggtgc atttaaagct gttcaccatg aacagatcga

1741 caatgtaaca gatgaacagc atgtaacacc taatagaaca ggtgaaacca gtaaaacaaa

1801 gcaactagaa catgaaattg aacacctgag acaacttgtt acagctcaac agtcacacat

1861 agacagcctg aaacaggcga tgctgcttat cgaatcaaag ctgccgacaa cacgggagcc

1921 agtgacgcct cccgtgggga aaaaatcatg gcaattctgg aagaaatagc gctgtgacgg

1981 aagatcactt cgcagaataa ataaatcctg gtgtccctgt tgataccggg aagccctggg

2041 ccaacttttg gcgaaaatga gacgttgatc ggcacgtaag aggttccaac tttcaccata

2101 atgaaataag atcactaccg ggcgtatttt ttgagttatc gagattttca ggagctaagg

2161 aagctaaaat ggagaaaaaa atcactggat ataccaccgt tgatatatcc caatggcatc

2221 gtaaagaaca ttttgaggca tttcagtcag ttgctcaatg tacctataac cagaccgttc

2281 agctggatat tacggccttt ttaaagaccg taaagaaaaa taagcacaag ttttatccgg

2341 cctttattca cattcttgcc cgcctgatga atgctcatcc ggaattccgt atggcaatga

2401 aagacggtga gctggtgata tgggatagtg ttcacccttg ttacaccgtt ttccatgagc

2461 aaactgaaac gttttcatcg ctctggagtg aataccacga cgatttccgg cagtttctac

2521 acatatattc gcaagatgtg gcgtgttacg gtgaaaacct ggcctatttc cctaaagggt

2581 ttattgagaa tatgtttttc gtctcagcca atccctgggt gagtttcacc agttttgatt

2641 taaacgtggc caatatggac aacttcttcg cccccgtttt caccatgggc aaatattata

2701 cgcaaggcga caaggtgctg atgccgctgg cgattcaggt tcatcatgcc gtctgtgatg

2761 gcttccatgt cggcagaatg cttaatgaat tacaacagta ctgcgatgag tggcagggcg

2821 gggcgtaatt tttttaaggc agttattggt gcccttaaac gcctggtgct acgcctgaat

2881 aagtgataat aagcggatga atggcagaaa ttcgaaagca aattcgaccc ggtcgtcggt

2941 tcagggcagg gtcgttaaat agccgcttat gtctattgct ggtttaccgg tttattgact

3001 accggaagca gtgtgaccgt gtgcttctca aatgcctgag gccagtttgc tcaggctctc

3061 ccatttaaat agaaacgcaa aaaggccatc cgtcaggatg gccttctgct taatttgatg

3121 cctggcagtt tatggcgggc gtcctgcccg ccaccctccg ggccgttgct tcgcaacgtt

3181 caaatccgct cccggcggat ttgtcctact caggagagcg ttcaccgaca aacaacagat

3241 aaaacgaaag gcccagtctt tcgactgagc ctttcgtttt atttgatgcc tggcagttcc

3301 ctactctcgc atggggagac cccacactac catcggcgct acggcgtttc acttctgagt

3361 tcggcatggg gtcaggtggg accaccgcgc tactgccgcc aggcaaattc tgttttatca

3421 gaccgcttct gcgttctgat ttaatctgta tcaggctgaa aatcttctct catccgccaa

3481 aacagccaag cttgcatgcc tgcaggtcga ctctagagga tccccgggta ccttacttcc

3541 cgggtggcgc gtcacgctcc acgctcagct gatctctggc agcgccccgt ttaacgccat

3601 gacgttctcg cagcgtcgcc tgcagttttg acagcggcgg cgcgtaaaga aagccaaacg

3661 agacacagcc ctctttgcct cgtaccgcat gatgcatgcg gtgcgccatg taaagcctgc

3721 gtaaataacc ccggcgcgga acatagcgga atggccagcg ctgatgtacc aggccatcat

3781 gcacaataaa atagagcagg ccgtagagcg tcatacccgc gccgatccac tgcaagggcc

3841 agacacccgt gctgcccaga taaatgagca aaatcgacag agcggcgaac actacggcat

3901 agaggtcatt aacctcaaac cagcctttat gcggttcatg atgtgacaga tgccagcccc

3961 aaccccagcc atgcataatg tatctgtgcg ccagtgcagc cgttatctcc attccgataa

4021 cagtgaccag aacaatcagg gcattccaaa tccacaacat

//

**pGlpF-CrtZ**

LOCUS pGlpF-CrtZ 4903 bp DNA circular SYN 19-MAR-1980

ACCESSION pGlpF-CrtZ

SOURCE Unknown.

ORGANISM Unknown

Unclassified.

REFERENCE 1 (bases 1 to 4903)

AUTHORS Self

JOURNAL Unpublished.

COMMENT SECID/File created by SciEd Central, Scientific & Educational Software

COMMENT This file is created by Vector NTI

http://www.invitrogen.com/

COMMENT ORIGDB|GenBank

COMMENT LSOWNER|

COMMENT VNTNAME|pGlpF-CrtZ|

FEATURES Location/Qualifiers

promoter complement (17..88)

/gene="M1-46"

/vntifkey="30"

/label=M1-46

CDS 218..574

/gene="ori"

/vntifkey="4"

/label=ori

CDS 598..1548

/gene="repA"

/vntifkey="4"

/label=repA

CDS 2169..2828

/gene="Cm"

/vntifkey="4"

/label=Cm

CDS complement (3533..4060)

/gene="crtZ"

/vntifkey="4"

/label=crtZ

CDS complement(4061..4903)

/vntifkey="4"

/label=GlpF

terminator 3080..3380

/vntifkey="43"

/label=rrnB

BASE COUNT 1481 a 1135 c 1103 g 1184 t

ORIGIN

1 agctgtttcc tggtttaaac cgaattggtg gggcgagagg ctcaattata tcaacgttgt

61 tatctcttgt caacaccgcc agagataagc gcctgtagtg ccatttaccc ccattcactg

121 ccagagccgt gagcgcagcg aactgaatgt cacgaaaaag acagcgactc aggtgcctga

181 tggtcggaga caaaaggaat attcagcgat ttgcccgagc ttgcgagggt gctacttaag

241 cctttagggt tttaaggtct gttttgtaga ggagcaaaca gcgtttgcga catccttttg

301 taatactgcg gaactgacta aagtagtgag ttatacacag ggctgggatc tattcttttt

361 atcttttttt attctttctt tattctataa attataacca cttgaatata aacaaaaaaa

421 acacacaaag gtctagcgga atttacagag ggtctagcag aatttacaag ttttccagca

481 aaggtctagc agaatttaca gatacccaca actcaaagga aaaggactag taattatcat

541 tgactagccc atctcaattg gtatagtgat taaaatcacc tagaccaatt gagatgtatg

601 tctgaattag ttgttttcaa agcaaatgaa ctagcgatta gtcgctatga cttaacggag

661 catgaaacca agctaatttt atgctgtgtg gcactactca accccacgat tgaaaaccct

721 acaaggaaag aacggacggt atcgttcact tataaccaat acgctcagat gatgaacatc

781 agtagggaaa atgcttatgg tgtattagct aaagcaacca gagagctgat gacgagaact

841 gtggaaatca ggaatccttt ggttaaaggc tttgagattt tccagtggac aaactatgcc

901 aagttctcaa gcgaaaaatt agaattagtt tttagtgaag agatattgcc ttatcttttc

961 cagttaaaaa aattcataaa atataatctg gaacatgtta agtcttttga aaacaaatac

1021 tctatgagga tttatgagtg gttattaaaa gaactaacac aaaagaaaac tcacaaggca

1081 aatatagaga ttagccttga tgaatttaag ttcatgttaa tgcttgaaaa taactaccat

1141 gagtttaaaa ggcttaacca atgggttttg aaaccaataa gtaaagattt aaacacttac

1201 agcaatatga aattggtggt tgataagcga ggccgcccga ctgatacgtt gattttccaa

1261 gttgaactag atagacaaat ggatctcgta accgaacttg agaacaacca gataaaaatg

1321 aatggtgaca aaataccaac aaccattaca tcagattcct acctacgtaa cggactaaga

1381 aaaacactac acgatgcttt aactgcaaaa attcagctca ccagttttga ggcaaaattt

1441 ttgagtgaca tgcaaagtaa gcatgatctc aatggttcgt tctcatggct cacgcaaaaa

1501 caacgaacca cactagagaa catactggct aaatacggaa ggatctgagg ttcttatggc

1561 tcttgtatct atcagtgaag catcaagact aacaaacaaa agtagaacaa ctgttcaccg

1621 ttagatatca aagggaaaac tgtccatatg cacagatgaa aacggtgtaa aaaagataga

1681 tacatcagag cttttacgag tttttggtgc atttaaagct gttcaccatg aacagatcga

1741 caatgtaaca gatgaacagc atgtaacacc taatagaaca ggtgaaacca gtaaaacaaa

1801 gcaactagaa catgaaattg aacacctgag acaacttgtt acagctcaac agtcacacat

1861 agacagcctg aaacaggcga tgctgcttat cgaatcaaag ctgccgacaa cacgggagcc

1921 agtgacgcct cccgtgggga aaaaatcatg gcaattctgg aagaaatagc gctgtgacgg

1981 aagatcactt cgcagaataa ataaatcctg gtgtccctgt tgataccggg aagccctggg

2041 ccaacttttg gcgaaaatga gacgttgatc ggcacgtaag aggttccaac tttcaccata

2101 atgaaataag atcactaccg ggcgtatttt ttgagttatc gagattttca ggagctaagg

2161 aagctaaaat ggagaaaaaa atcactggat ataccaccgt tgatatatcc caatggcatc

2221 gtaaagaaca ttttgaggca tttcagtcag ttgctcaatg tacctataac cagaccgttc

2281 agctggatat tacggccttt ttaaagaccg taaagaaaaa taagcacaag ttttatccgg

2341 cctttattca cattcttgcc cgcctgatga atgctcatcc ggaattccgt atggcaatga

2401 aagacggtga gctggtgata tgggatagtg ttcacccttg ttacaccgtt ttccatgagc

2461 aaactgaaac gttttcatcg ctctggagtg aataccacga cgatttccgg cagtttctac

2521 acatatattc gcaagatgtg gcgtgttacg gtgaaaacct ggcctatttc cctaaagggt

2581 ttattgagaa tatgtttttc gtctcagcca atccctgggt gagtttcacc agttttgatt

2641 taaacgtggc caatatggac aacttcttcg cccccgtttt caccatgggc aaatattata

2701 cgcaaggcga caaggtgctg atgccgctgg cgattcaggt tcatcatgcc gtctgtgatg

2761 gcttccatgt cggcagaatg cttaatgaat tacaacagta ctgcgatgag tggcagggcg

2821 gggcgtaatt tttttaaggc agttattggt gcccttaaac gcctggtgct acgcctgaat

2881 aagtgataat aagcggatga atggcagaaa ttcgaaagca aattcgaccc ggtcgtcggt

2941 tcagggcagg gtcgttaaat agccgcttat gtctattgct ggtttaccgg tttattgact

3001 accggaagca gtgtgaccgt gtgcttctca aatgcctgag gccagtttgc tcaggctctc

3061 ccatttaaat agaaacgcaa aaaggccatc cgtcaggatg gccttctgct taatttgatg

3121 cctggcagtt tatggcgggc gtcctgcccg ccaccctccg ggccgttgct tcgcaacgtt

3181 caaatccgct cccggcggat ttgtcctact caggagagcg ttcaccgaca aacaacagat

3241 aaaacgaaag gcccagtctt tcgactgagc ctttcgtttt atttgatgcc tggcagttcc

3301 ctactctcgc atggggagac cccacactac catcggcgct acggcgtttc acttctgagt

3361 tcggcatggg gtcaggtggg accaccgcgc tactgccgcc aggcaaattc tgttttatca

3421 gaccgcttct gcgttctgat ttaatctgta tcaggctgaa aatcttctct catccgccaa

3481 aacagccaag cttgcatgcc tgcaggtcga ctctagagga tccccgggta ccttacttcc

3541 cgggtggcgc gtcacgctcc acgctcagct gatctctggc agcgccccgt ttaacgccat

3601 gacgttctcg cagcgtcgcc tgcagttttg acagcggcgg cgcgtaaaga aagccaaacg

3661 agacacagcc ctctttgcct cgtaccgcat gatgcatgcg gtgcgccatg taaagcctgc

3721 gtaaataacc ccggcgcgga acatagcgga atggccagcg ctgatgtacc aggccatcat

3781 gcacaataaa atagagcagg ccgtagagcg tcatacccgc gccgatccac tgcaagggcc

3841 agacacccgt gctgcccaga taaatgagca aaatcgacag agcggcgaac actacggcat

3901 agaggtcatt aacctcaaac cagcctttat gcggttcatg atgtgacaga tgccagcccc

3961 aaccccagcc atgcataatg tatctgtgcg ccagtgcagc cgttatctcc attccgataa

4021 cagtgaccag aacaatcagg gcattccaaa tccacaacat cagcgaagct ttttgttctg

4081 aaggagttgt ggtttccttt tcttccacaa cacagatatc gcaaggcaaa tggcgaccaa

4141 tcagtttgcg gtaggcaaat gcacctacaa tcgcgccaac gatagggccg aaaagcggca

4201 ccaggaagta aggaatgtct ctgccgccgg taaaggcgac attgccccag cccgccagcc

4261 aggcaaagac tttcggaccg aagtcacgcg ctgggttcat ggcaaaacct gtcaatgggc

4321 ccatagatgc gccaatgacc gcaatcagta gaccaatcag caagggagcc aaagggccgc

4381 gtggtacacc gttgccatcg tccgttaacg ccaggatcag ccccatcaga atagcggtaa

4441 tcaccatctc aactgcgaaa gcctgcacaa aattgatatg aggattaggg taagtagaga

4501 aagtgccagc cagatcaaca ctttcaacgc tgccgcgaac aatgtgatga gtctgctcga

4561 agtcgaaaaa taaattgtag taaagcccgt aaactaaagc cgcagcacag aaagcgccgg

4621 caacttgtga aacgataaaa ggaataactt tgcgcttgtc gaaacaggca aacagccaca

4681 atgcaatggt aacagcggga ttaagatgcg cgccggaaac ccctgcggtc aggtagatgg

4741 ccattgccac ccccagtccc caaatgacac tgatttccca ctgaccaaaa gacgcaccag

4801 cgacttttag tgctgcaacg caacccacac cgaagaaaat caacaacccg gtaccgagga

4861 attcagcaat gcactggcct ttcaaggttg atgtttgact cat

//

**pGlpF-CrtW/GlpF-CrtZ**

LOCUS pGlpF-CrtW/GlpF-CrtZ 6497 bp DNA circular SYN 19-MAR-1980

ACCESSION pGlpF-CrtW/GlpF-CrtZ

SOURCE Unknown.

ORGANISM Unknown

Unclassified.

REFERENCE 1 (bases 1 to 6497)

AUTHORS Self

JOURNAL Unpublished.

COMMENT SECID/File created by SciEd Central, Scientific & Educational Software

COMMENT This file is created by Vector NTI

http://www.invitrogen.com/

COMMENT ORIGDB|GenBank

COMMENT LSOWNER|

COMMENT VNTNAME|pGlpF-CrtW/GlpF-|

FEATURES Location/Qualifiers

CDS complement (1..735)

/gene="crtW"

/vntifkey="4"

/label=crtW

CDS complement (736..1578)

/gene="GlpF"

/vntifkey="4"

/label=GlpF

promoter complement (1595..1666)

/gene="M1-46"

/vntifkey="30"

/label=M1-46

rep_origin 1796..2152

/gene="ori"

/vntifkey="33"

/label=ori

CDS 2176..3126

/gene="repA"

/vntifkey="4"

/label=repA

CDS 3747..4406

/gene="Cm"

/vntifkey="4"

/label=Cm

terminator 4658..4958

/gene="rrnB"

/vntifkey="43"

/label=rrnB

CDS complement (5111..5638)

/gene="crtZ"

/vntifkey="4"

/label=crtZ

CDS complement (5639..6481)

/gene="GlpF"

/vntifkey="4"

/label=GlpF

BASE COUNT 1934 a 1581 c 1531 g 1451 t

ORIGIN

1 tcacgattca ccacgccaca gacgccacca cggacgccac ggggtcagat gatgttcatg

61 gtgacgaccc agatggaagc acgtcagcag agacagaacc ggaccgtaac cggaactacg

121 tgcgtgatgt gcgtctgcaa acggttgatc ggtgtgacga tgcggcagcc acgtgccgaa

181 ggtaaacatc tgcagtgcgc tcagcagtgc cggtgctgcc cagaaggtca gcagatttgc

241 cggacgtgca cccagaccaa acagtgcgat cagaaccaga gccgtcagga ctgccatttc

301 gcgccagccg aagtaggtac ggaaaaagtt caggaaccac ggcagaaaag cacgcggagc

361 cggtgcataa aagtccgggt catctgccgt acccggagct gcgtgatgag cgtgatgtgc

421 ggttttcagg cgatcgaaac gaaaaccggc atacaggccc agggtcaggc ggcccacggc

481 cgcgttcaga cgcggacgac ccggcgccag cgaaccgtgc atggcatcat gcgcaacaat

541 aaacagaccc actgacagcc acgtctgcac agcgacgatt gccggaacaa tcaccagaga

601 gctggtgccc caacgatgaa agtagacacc ataaacatgc agggagcccc agccagcaac

661 aatcatacca gccagggtca ggccaatcca ggtttgacgc gggacaatac gcggttctgc

721 gactgcggcg gtcatcagcg aagctttttg ttctgaagga gttgtggttt ccttttcttc

781 cacaacacag atatcgcaag gcaaatggcg accaatcagt ttgcggtagg caaatgcacc

841 tacaatcgcg ccaacgatag ggccgaaaag cggcaccagg aagtaaggaa tgtctctgcc

901 gccggtaaag gcgacattgc cccagcccgc cagccaggca aagactttcg gaccgaagtc

961 acgcgctggg ttcatggcaa aacctgtcaa tgggcccata gatgcgccaa tgaccgcaat

1021 cagtagacca atcagcaagg gagccaaagg gccgcgtggt acaccgttgc catcgtccgt

1081 taacgccagg atcagcccca tcagaatagc ggtaatcacc atctcaactg cgaaagcctg

1141 cacaaaattg atatgaggat tagggtaagt agagaaagtg ccagccagat caacactttc

1201 aacgctgccg cgaacaatgt gatgagtctg ctcgaagtcg aaaaataaat tgtagtaaag

1261 cccgtaaact aaagccgcag cacagaaagc gccggcaact tgtgaaacga taaaaggaat

1321 aactttgcgc ttgtcgaaac aggcaaacag ccacaatgca atggtaacag cgggattaag

1381 atgcgcgccg gaaacccctg cggtcaggta gatggccatt gccaccccca gtccccaaat

1441 gacactgatt tcccactgac caaaagacgc accagcgact tttagtgctg caacgcaacc

1501 cacaccgaag aaaatcaaca acccggtacc gaggaattca gcaatgcact ggcctttcaa

1561 ggttgatgtt tgactcatag ctgtttcctg gtttaaaccg aattggtggg gcgagaggct

1621 caattatatc aacgttgtta tctcttgtca acaccgccag agataagcgc ctgtagtgcc

1681 atttaccccc attcactgcc agagccgtga gcgcagcgaa ctgaatgtca cgaaaaagac

1741 agcgactcag gtgcctgatg gtcggagaca aaaggaatat tcagcgattt gcccgagctt

1801 gcgagggtgc tacttaagcc tttagggttt taaggtctgt tttgtagagg agcaaacagc

1861 gtttgcgaca tccttttgta atactgcgga actgactaaa gtagtgagtt atacacaggg

1921 ctgggatcta ttctttttat ctttttttat tctttcttta ttctataaat tataaccact

1981 tgaatataaa caaaaaaaac acacaaaggt ctagcggaat ttacagaggg tctagcagaa

2041 tttacaagtt ttccagcaaa ggtctagcag aatttacaga tacccacaac tcaaaggaaa

2101 aggactagta attatcattg actagcccat ctcaattggt atagtgatta aaatcaccta

2161 gaccaattga gatgtatgtc tgaattagtt gttttcaaag caaatgaact agcgattagt

2221 cgctatgact taacggagca tgaaaccaag ctaattttat gctgtgtggc actactcaac

2281 cccacgattg aaaaccctac aaggaaagaa cggacggtat cgttcactta taaccaatac

2341 gctcagatga tgaacatcag tagggaaaat gcttatggtg tattagctaa agcaaccaga

2401 gagctgatga cgagaactgt ggaaatcagg aatcctttgg ttaaaggctt tgagattttc

2461 cagtggacaa actatgccaa gttctcaagc gaaaaattag aattagtttt tagtgaagag

2521 atattgcctt atcttttcca gttaaaaaaa ttcataaaat ataatctgga acatgttaag

2581 tcttttgaaa acaaatactc tatgaggatt tatgagtggt tattaaaaga actaacacaa

2641 aagaaaactc acaaggcaaa tatagagatt agccttgatg aatttaagtt catgttaatg

2701 cttgaaaata actaccatga gtttaaaagg cttaaccaat gggttttgaa accaataagt

2761 aaagatttaa acacttacag caatatgaaa ttggtggttg ataagcgagg ccgcccgact

2821 gatacgttga ttttccaagt tgaactagat agacaaatgg atctcgtaac cgaacttgag

2881 aacaaccaga taaaaatgaa tggtgacaaa ataccaacaa ccattacatc agattcctac

2941 ctacgtaacg gactaagaaa aacactacac gatgctttaa ctgcaaaaat tcagctcacc

3001 agttttgagg caaaattttt gagtgacatg caaagtaagc atgatctcaa tggttcgttc

3061 tcatggctca cgcaaaaaca acgaaccaca ctagagaaca tactggctaa atacggaagg

3121 atctgaggtt cttatggctc ttgtatctat cagtgaagca tcaagactaa caaacaaaag

3181 tagaacaact gttcaccgtt agatatcaaa gggaaaactg tccatatgca cagatgaaaa

3241 cggtgtaaaa aagatagata catcagagct tttacgagtt tttggtgcat ttaaagctgt

3301 tcaccatgaa cagatcgaca atgtaacaga tgaacagcat gtaacaccta atagaacagg

3361 tgaaaccagt aaaacaaagc aactagaaca tgaaattgaa cacctgagac aacttgttac

3421 agctcaacag tcacacatag acagcctgaa acaggcgatg ctgcttatcg aatcaaagct

3481 gccgacaaca cgggagccag tgacgcctcc cgtggggaaa aaatcatggc aattctggaa

3541 gaaatagcgc tgtgacggaa gatcacttcg cagaataaat aaatcctggt gtccctgttg

3601 ataccgggaa gccctgggcc aacttttggc gaaaatgaga cgttgatcgg cacgtaagag

3661 gttccaactt tcaccataat gaaataagat cactaccggg cgtatttttt gagttatcga

3721 gattttcagg agctaaggaa gctaaaatgg agaaaaaaat cactggatat accaccgttg

3781 atatatccca atggcatcgt aaagaacatt ttgaggcatt tcagtcagtt gctcaatgta

3841 cctataacca gaccgttcag ctggatatta cggccttttt aaagaccgta aagaaaaata

3901 agcacaagtt ttatccggcc tttattcaca ttcttgcccg cctgatgaat gctcatccgg

3961 aattccgtat ggcaatgaaa gacggtgagc tggtgatatg ggatagtgtt cacccttgtt

4021 acaccgtttt ccatgagcaa actgaaacgt tttcatcgct ctggagtgaa taccacgacg

4081 atttccggca gtttctacac atatattcgc aagatgtggc gtgttacggt gaaaacctgg

4141 cctatttccc taaagggttt attgagaata tgtttttcgt ctcagccaat ccctgggtga

4201 gtttcaccag ttttgattta aacgtggcca atatggacaa cttcttcgcc cccgttttca

4261 ccatgggcaa atattatacg caaggcgaca aggtgctgat gccgctggcg attcaggttc

4321 atcatgccgt ctgtgatggc ttccatgtcg gcagaatgct taatgaatta caacagtact

4381 gcgatgagtg gcagggcggg gcgtaatttt tttaaggcag ttattggtgc ccttaaacgc

4441 ctggtgctac gcctgaataa gtgataataa gcggatgaat ggcagaaatt cgaaagcaaa

4501 ttcgacccgg tcgtcggttc agggcagggt cgttaaatag ccgcttatgt ctattgctgg

4561 tttaccggtt tattgactac cggaagcagt gtgaccgtgt gcttctcaaa tgcctgaggc

4621 cagtttgctc aggctctccc atttaaatag aaacgcaaaa aggccatccg tcaggatggc

4681 cttctgctta atttgatgcc tggcagttta tggcgggcgt cctgcccgcc accctccggg

4741 ccgttgcttc gcaacgttca aatccgctcc cggcggattt gtcctactca ggagagcgtt

4801 caccgacaaa caacagataa aacgaaaggc ccagtctttc gactgagcct ttcgttttat

4861 ttgatgcctg gcagttccct actctcgcat ggggagaccc cacactacca tcggcgctac

4921 ggcgtttcac ttctgagttc ggcatggggt caggtgggac caccgcgcta ctgccgccag

4981 gcaaattctg ttttatcaga ccgcttctgc gttctgattt aatctgtatc aggctgaaaa

5041 tcttctctca tccgccaaaa cagccaagct tgcatgcctg caggtcgact ctagaggatc

5101 cccgggtacc ttacttcccg ggtggcgcgt cacgctccac gctcagctga tctctggcag

5161 cgccccgttt aacgccatga cgttctcgca gcgtcgcctg cagttttgac agcggcggcg

5221 cgtaaagaaa gccaaacgag acacagccct ctttgcctcg taccgcatga tgcatgcggt

5281 gcgccatgta aagcctgcgt aaataacccc ggcgcggaac atagcggaat ggccagcgct

5341 gatgtaccag gccatcatgc acaataaaat agagcaggcc gtagagcgtc atacccgcgc

5401 cgatccactg caagggccag acacccgtgc tgcccagata aatgagcaaa atcgacagag

5461 cggcgaacac tacggcatag aggtcattaa cctcaaacca gcctttatgc ggttcatgat

5521 gtgacagatg ccagccccaa ccccagccat gcataatgta tctgtgcgcc agtgcagccg

5581 ttatctccat tccgataaca gtgaccagaa caatcagggc attccaaatc cacaacatca

5641 gcgaagcttt ttgttctgaa ggagttgtgg tttccttttc ttccacaaca cagatatcgc

5701 aaggcaaatg gcgaccaatc agtttgcggt aggcaaatgc acctacaatc gcgccaacga

5761 tagggccgaa aagcggcacc aggaagtaag gaatgtctct gccgccggta aaggcgacat

5821 tgccccagcc cgccagccag gcaaagactt tcggaccgaa gtcacgcgct gggttcatgg

5881 caaaacctgt caatgggccc atagatgcgc caatgaccgc aatcagtaga ccaatcagca

5941 agggagccaa agggccgcgt ggtacaccgt tgccatcgtc cgttaacgcc aggatcagcc

6001 ccatcagaat agcggtaatc accatctcaa ctgcgaaagc ctgcacaaaa ttgatatgag

6061 gattagggta agtagagaaa gtgccagcca gatcaacact ttcaacgctg ccgcgaacaa

6121 tgtgatgagt ctgctcgaag tcgaaaaata aattgtagta aagcccgtaa actaaagccg

6181 cagcacagaa agcgccggca acttgtgaaa cgataaaagg aataactttg cgcttgtcga

6241 aacaggcaaa cagccacaat gcaatggtaa cagcgggatt aagatgcgcg ccggaaaccc

6301 ctgcggtcag gtagatggcc attgccaccc ccagtcccca aatgacactg atttcccact

6361 gaccaaaaga cgcaccagcg acttttagtg ctgcaacgca acccacaccg aagaaaatca

6421 acaacccggt accgaggaat tcagcaatgc actggccttt caaggttgat gtttgactca

6481 tagctgtttc ctggttt

//

**pCrtW-CrtZ**

LOCUS pCrtW-CrtZ 4816 bp DNA circular SYN 19-MAR-1980

ACCESSION pCrtW-CrtZ

SOURCE Unknown.

ORGANISM Unknown

Unclassified.

REFERENCE 1 (bases 1 to 4816)

AUTHORS Self

JOURNAL Unpublished.

COMMENT SECID/File created by SciEd Central, Scientific & Educational Software

COMMENT This file is created by Vector NTI

http://www.invitrogen.com/

COMMENT ORIGDB|GenBank

COMMENT LSOWNER|

COMMENT VNTNAME|pCrtW-CrtZ|

FEATURES Location/Qualifiers

rep_origin 130..486

/gene="ori"

/vntifkey="33"

/label=ori

CDS 510..1460

/gene="repA"

/vntifkey="4"

/label=repA

CDS 2081..2740

/gene="Cm"

/vntifkey="4"

/label=Cm

terminator 2992..3292

/gene="rrnB"

/vntifkey="43"

/label=rrnB

CDS complement (3445..3972)

/gene="crtZ"

/vntifkey="4"

/label=crtZ

misc_feature complement (3973..3996)

/gene="linker-2"

/vntifkey="21"

/label=linker-2

CDS complement (3997..4728)

/gene="crtW"

/vntifkey="4"

/label=crtW

promoter complement(4745..4816)

/vntifkey="30"

/label=M1-46

BASE COUNT 1424 a 1130 c 1123 g 1139 t

ORIGIN

1 gcgcctgtag tgccatttac ccccattcac tgccagagcc gtgagcgcag cgaactgaat

61 gtcacgaaaa agacagcgac tcaggtgcct gatggtcgga gacaaaagga atattcagcg

121 atttgcccga gcttgcgagg gtgctactta agcctttagg gttttaaggt ctgttttgta

181 gaggagcaaa cagcgtttgc gacatccttt tgtaatactg cggaactgac taaagtagtg

241 agttatacac agggctggga tctattcttt ttatcttttt ttattctttc tttattctat

301 aaattataac cacttgaata taaacaaaaa aaacacacaa aggtctagcg gaatttacag

361 agggtctagc agaatttaca agttttccag caaaggtcta gcagaattta cagataccca

421 caactcaaag gaaaaggact agtaattatc attgactagc ccatctcaat tggtatagtg

481 attaaaatca cctagaccaa ttgagatgta tgtctgaatt agttgttttc aaagcaaatg

541 aactagcgat tagtcgctat gacttaacgg agcatgaaac caagctaatt ttatgctgtg

601 tggcactact caaccccacg attgaaaacc ctacaaggaa agaacggacg gtatcgttca

661 cttataacca atacgctcag atgatgaaca tcagtaggga aaatgcttat ggtgtattag

721 ctaaagcaac cagagagctg atgacgagaa ctgtggaaat caggaatcct ttggttaaag

781 gctttgagat tttccagtgg acaaactatg ccaagttctc aagcgaaaaa ttagaattag

841 tttttagtga agagatattg ccttatcttt tccagttaaa aaaattcata aaatataatc

901 tggaacatgt taagtctttt gaaaacaaat actctatgag gatttatgag tggttattaa

961 aagaactaac acaaaagaaa actcacaagg caaatataga gattagcctt gatgaattta

1021 agttcatgtt aatgcttgaa aataactacc atgagtttaa aaggcttaac caatgggttt

1081 tgaaaccaat aagtaaagat ttaaacactt acagcaatat gaaattggtg gttgataagc

1141 gaggccgccc gactgatacg ttgattttcc aagttgaact agatagacaa atggatctcg

1201 taaccgaact tgagaacaac cagataaaaa tgaatggtga caaaatacca acaaccatta

1261 catcagattc ctacctacgt aacggactaa gaaaaacact acacgatgct ttaactgcaa

1321 aaattcagct caccagtttt gaggcaaaat ttttgagtga catgcaaagt aagcatgatc

1381 tcaatggttc gttctcatgg ctcacgcaaa aacaacgaac cacactagag aacatactgg

1441 ctaaatacgg aaggatctga ggttcttatg gctcttgtat ctatcagtga agcatcaaga

1501 ctaacaaaca aaagtagaac aactgttcac cgttagatat caaagggaaa actgtccata

1561 tgcacagatg aaaacggtgt aaaaaagata gatacatcag agcttttacg agtttttggt

1621 gcatttaaag ctgttcacca tgaacagatc gacaatgtaa cagatgaaca gcatgtaaca

1681 cctaatagaa caggtgaaac cagtaaaaca aagcaactag aacatgaaat tgaacacctg

1741 agacaacttg ttacagctca acagtcacac atagacagcc tgaaacaggc gatgctgctt

1801 atcgaatcaa agctgccgac aacacgggag ccagtgacgc ctcccgtggg gaaaaaatca

1861 tggcaattct ggaagaaata gcgctgtgac ggaagatcac ttcgcagaat aaataaatcc

1921 tggtgtccct gttgataccg ggaagccctg ggccaacttt tggcgaaaat gagacgttga

1981 tcggcacgta agaggttcca actttcacca taatgaaata agatcactac cgggcgtatt

2041 ttttgagtta tcgagatttt caggagctaa ggaagctaaa atggagaaaa aaatcactgg

2101 atataccacc gttgatatat cccaatggca tcgtaaagaa cattttgagg catttcagtc

2161 agttgctcaa tgtacctata accagaccgt tcagctggat attacggcct ttttaaagac

2221 cgtaaagaaa aataagcaca agttttatcc ggcctttatt cacattcttg cccgcctgat

2281 gaatgctcat ccggaattcc gtatggcaat gaaagacggt gagctggtga tatgggatag

2341 tgttcaccct tgttacaccg ttttccatga gcaaactgaa acgttttcat cgctctggag

2401 tgaataccac gacgatttcc ggcagtttct acacatatat tcgcaagatg tggcgtgtta

2461 cggtgaaaac ctggcctatt tccctaaagg gtttattgag aatatgtttt tcgtctcagc

2521 caatccctgg gtgagtttca ccagttttga tttaaacgtg gccaatatgg acaacttctt

2581 cgcccccgtt ttcaccatgg gcaaatatta tacgcaaggc gacaaggtgc tgatgccgct

2641 ggcgattcag gttcatcatg ccgtctgtga tggcttccat gtcggcagaa tgcttaatga

2701 attacaacag tactgcgatg agtggcaggg cggggcgtaa tttttttaag gcagttattg

2761 gtgcccttaa acgcctggtg ctacgcctga ataagtgata ataagcggat gaatggcaga

2821 aattcgaaag caaattcgac ccggtcgtcg gttcagggca gggtcgttaa atagccgctt

2881 atgtctattg ctggtttacc ggtttattga ctaccggaag cagtgtgacc gtgtgcttct

2941 caaatgcctg aggccagttt gctcaggctc tcccatttaa atagaaacgc aaaaaggcca

3001 tccgtcagga tggccttctg cttaatttga tgcctggcag tttatggcgg gcgtcctgcc

3061 cgccaccctc cgggccgttg cttcgcaacg ttcaaatccg ctcccggcgg atttgtccta

3121 ctcaggagag cgttcaccga caaacaacag ataaaacgaa aggcccagtc tttcgactga

3181 gcctttcgtt ttatttgatg cctggcagtt ccctactctc gcatggggag accccacact

3241 accatcggcg ctacggcgtt tcacttctga gttcggcatg gggtcaggtg ggaccaccgc

3301 gctactgccg ccaggcaaat tctgttttat cagaccgctt ctgcgttctg atttaatctg

3361 tatcaggctg aaaatcttct ctcatccgcc aaaacagcca agcttgcatg cctgcaggtc

3421 gactctagag gatccccggg taccttactt cccgggtggc gcgtcacgct ccacgctcag

3481 ctgatctctg gcagcgcccc gtttaacgcc atgacgttct cgcagcgtcg cctgcagttt

3541 tgacagcggc ggcgcgtaaa gaaagccaaa cgagacacag ccctctttgc ctcgtaccgc

3601 atgatgcatg cggtgcgcca tgtaaagcct gcgtaaataa ccccggcgcg gaacatagcg

3661 gaatggccag cgctgatgta ccaggccatc atgcacaata aaatagagca ggccgtagag

3721 cgtcataccc gcgccgatcc actgcaaggg ccagacaccc gtgctgccca gataaatgag

3781 caaaatcgac agagcggcga acactacggc atagaggtca ttaacctcaa accagccttt

3841 atgcggttca tgatgtgaca gatgccagcc ccaaccccag ccatgcataa tgtatctgtg

3901 cgccagtgca gccgttatct ccattccgat aacagtgacc agaacaatca gggcattcca

3961 aatccacaac ataatacagc ctggtgctta agttcgcgat tcaccacgcc acagacgcca

4021 ccacggacgc cacggggtca gatgatgttc atggtgacga cccagatgga agcacgtcag

4081 cagagacaga accggaccgt aaccggaact acgtgcgtga tgtgcgtctg caaacggttg

4141 atcggtgtga cgatgcggca gccacgtgcc gaaggtaaac atctgcagtg cgctcagcag

4201 tgccggtgct gcccagaagg tcagcagatt tgccggacgt gcacccagac caaacagtgc

4261 gatcagaacc agagccgtca ggactgccat ttcgcgccag ccgaagtagg tacggaaaaa

4321 gttcaggaac cacggcagaa aagcacgcgg agccggtgca taaaagtccg ggtcatctgc

4381 cgtacccgga gctgcgtgat gagcgtgatg tgcggttttc aggcgatcga aacgaaaacc

4441 ggcatacagg cccagggtca ggcggcccac ggccgcgttc agacgcggac gacccggcgc

4501 cagcgaaccg tgcatggcat catgcgcaac aataaacaga cccactgaca gccacgtctg

4561 cacagcgacg attgccggaa caatcaccag agagctggtg ccccaacgat gaaagtagac

4621 accataaaca tgcagggagc cccagccagc aacaatcata ccagccaggg tcaggccaat

4681 ccaggtttga cgcgggacaa tacgcggttc tgcgactgcg gcggtcatag ctgtttcctg

4741 gtttaaaccg aattggtggg gcgagaggct caattatatc aacgttgtta tctcttgtca

4801 acaccgccag agataa

//

**pGlpF-CrtW-CrtZ**

LOCUS pGlpF-CrtW-CrtZ 5659 bp DNA circular SYN 19-MAR-1980

DEFINITION Ligation of inverted SacI+RBS+crtZ+KpnI* into pYL002*.

ACCESSION pGlpF-CrtW-CrtZ

SOURCE Unknown.

ORGANISM Unknown

Unclassified.

REFERENCE 1 (bases 1 to 5659)

AUTHORS Self

JOURNAL Unpublished.

COMMENT SECID/File created by SciEd Central, Scientific & Educational Software

COMMENT This file is created by Vector NTI

http://www.invitrogen.com/

COMMENT ORIGDB|GenBank

COMMENT LSOWNER|

COMMENT VNTNAME|pGlpF-CrtW-CrtZ|

FEATURES Location/Qualifiers

rep_origin 130..486

/gene="ori"

/vntifkey="33"

/label=ori

CDS 510..1460

/gene="repA"

/vntifkey="4"

/label=repA

CDS 2081..2740

/gene="Cm"

/vntifkey="4"

/label=Cm

terminator complement(2992..3292)

/gene="rrnB"

/vntifkey="43"

/label=rrnB

CDS complement (3445..3972)

/gene="crtZ"

/vntifkey="4"

/label=crtZ

misc_feature 3973..3996

/gene="linker-2"

/vntifkey="21"

/label=linker-2

CDS complement (3997..4728)

/gene="crtW"

/vntifkey="4"

/label=crtW

promoter complement (5588..5659)

/gene="'M1-46"

/vntifkey="30"

/label=M1-46

CDS complement(4729..5571)

/vntifkey="4"

/label=GlpF

BASE COUNT 1682 a 1357 c 1327 g 1293 t

ORIGIN

1 gcgcctgtag tgccatttac ccccattcac tgccagagcc gtgagcgcag cgaactgaat

61 gtcacgaaaa agacagcgac tcaggtgcct gatggtcgga gacaaaagga atattcagcg

121 atttgcccga gcttgcgagg gtgctactta agcctttagg gttttaaggt ctgttttgta

181 gaggagcaaa cagcgtttgc gacatccttt tgtaatactg cggaactgac taaagtagtg

241 agttatacac agggctggga tctattcttt ttatcttttt ttattctttc tttattctat

301 aaattataac cacttgaata taaacaaaaa aaacacacaa aggtctagcg gaatttacag

361 agggtctagc agaatttaca agttttccag caaaggtcta gcagaattta cagataccca

421 caactcaaag gaaaaggact agtaattatc attgactagc ccatctcaat tggtatagtg

481 attaaaatca cctagaccaa ttgagatgta tgtctgaatt agttgttttc aaagcaaatg

541 aactagcgat tagtcgctat gacttaacgg agcatgaaac caagctaatt ttatgctgtg

601 tggcactact caaccccacg attgaaaacc ctacaaggaa agaacggacg gtatcgttca

661 cttataacca atacgctcag atgatgaaca tcagtaggga aaatgcttat ggtgtattag

721 ctaaagcaac cagagagctg atgacgagaa ctgtggaaat caggaatcct ttggttaaag

781 gctttgagat tttccagtgg acaaactatg ccaagttctc aagcgaaaaa ttagaattag

841 tttttagtga agagatattg ccttatcttt tccagttaaa aaaattcata aaatataatc

901 tggaacatgt taagtctttt gaaaacaaat actctatgag gatttatgag tggttattaa

961 aagaactaac acaaaagaaa actcacaagg caaatataga gattagcctt gatgaattta

1021 agttcatgtt aatgcttgaa aataactacc atgagtttaa aaggcttaac caatgggttt

1081 tgaaaccaat aagtaaagat ttaaacactt acagcaatat gaaattggtg gttgataagc

1141 gaggccgccc gactgatacg ttgattttcc aagttgaact agatagacaa atggatctcg

1201 taaccgaact tgagaacaac cagataaaaa tgaatggtga caaaatacca acaaccatta

1261 catcagattc ctacctacgt aacggactaa gaaaaacact acacgatgct ttaactgcaa

1321 aaattcagct caccagtttt gaggcaaaat ttttgagtga catgcaaagt aagcatgatc

1381 tcaatggttc gttctcatgg ctcacgcaaa aacaacgaac cacactagag aacatactgg

1441 ctaaatacgg aaggatctga ggttcttatg gctcttgtat ctatcagtga agcatcaaga

1501 ctaacaaaca aaagtagaac aactgttcac cgttagatat caaagggaaa actgtccata

1561 tgcacagatg aaaacggtgt aaaaaagata gatacatcag agcttttacg agtttttggt

1621 gcatttaaag ctgttcacca tgaacagatc gacaatgtaa cagatgaaca gcatgtaaca

1681 cctaatagaa caggtgaaac cagtaaaaca aagcaactag aacatgaaat tgaacacctg

1741 agacaacttg ttacagctca acagtcacac atagacagcc tgaaacaggc gatgctgctt

1801 atcgaatcaa agctgccgac aacacgggag ccagtgacgc ctcccgtggg gaaaaaatca

1861 tggcaattct ggaagaaata gcgctgtgac ggaagatcac ttcgcagaat aaataaatcc

1921 tggtgtccct gttgataccg ggaagccctg ggccaacttt tggcgaaaat gagacgttga

1981 tcggcacgta agaggttcca actttcacca taatgaaata agatcactac cgggcgtatt

2041 ttttgagtta tcgagatttt caggagctaa ggaagctaaa atggagaaaa aaatcactgg

2101 atataccacc gttgatatat cccaatggca tcgtaaagaa cattttgagg catttcagtc

2161 agttgctcaa tgtacctata accagaccgt tcagctggat attacggcct ttttaaagac

2221 cgtaaagaaa aataagcaca agttttatcc ggcctttatt cacattcttg cccgcctgat

2281 gaatgctcat ccggaattcc gtatggcaat gaaagacggt gagctggtga tatgggatag

2341 tgttcaccct tgttacaccg ttttccatga gcaaactgaa acgttttcat cgctctggag

2401 tgaataccac gacgatttcc ggcagtttct acacatatat tcgcaagatg tggcgtgtta

2461 cggtgaaaac ctggcctatt tccctaaagg gtttattgag aatatgtttt tcgtctcagc

2521 caatccctgg gtgagtttca ccagttttga tttaaacgtg gccaatatgg acaacttctt

2581 cgcccccgtt ttcaccatgg gcaaatatta tacgcaaggc gacaaggtgc tgatgccgct

2641 ggcgattcag gttcatcatg ccgtctgtga tggcttccat gtcggcagaa tgcttaatga

2701 attacaacag tactgcgatg agtggcaggg cggggcgtaa tttttttaag gcagttattg

2761 gtgcccttaa acgcctggtg ctacgcctga ataagtgata ataagcggat gaatggcaga

2821 aattcgaaag caaattcgac ccggtcgtcg gttcagggca gggtcgttaa atagccgctt

2881 atgtctattg ctggtttacc ggtttattga ctaccggaag cagtgtgacc gtgtgcttct

2941 caaatgcctg aggccagttt gctcaggctc tcccatttaa atagaaacgc aaaaaggcca

3001 tccgtcagga tggccttctg cttaatttga tgcctggcag tttatggcgg gcgtcctgcc

3061 cgccaccctc cgggccgttg cttcgcaacg ttcaaatccg ctcccggcgg atttgtccta

3121 ctcaggagag cgttcaccga caaacaacag ataaaacgaa aggcccagtc tttcgactga

3181 gcctttcgtt ttatttgatg cctggcagtt ccctactctc gcatggggag accccacact

3241 accatcggcg ctacggcgtt tcacttctga gttcggcatg gggtcaggtg ggaccaccgc

3301 gctactgccg ccaggcaaat tctgttttat cagaccgctt ctgcgttctg atttaatctg

3361 tatcaggctg aaaatcttct ctcatccgcc aaaacagcca agcttgcatg cctgcaggtc

3421 gactctagag gatccccggg taccttactt cccgggtggc gcgtcacgct ccacgctcag

3481 ctgatctctg gcagcgcccc gtttaacgcc atgacgttct cgcagcgtcg cctgcagttt

3541 tgacagcggc ggcgcgtaaa gaaagccaaa cgagacacag ccctctttgc ctcgtaccgc

3601 atgatgcatg cggtgcgcca tgtaaagcct gcgtaaataa ccccggcgcg gaacatagcg

3661 gaatggccag cgctgatgta ccaggccatc atgcacaata aaatagagca ggccgtagag

3721 cgtcataccc gcgccgatcc actgcaaggg ccagacaccc gtgctgccca gataaatgag

3781 caaaatcgac agagcggcga acactacggc atagaggtca ttaacctcaa accagccttt

3841 atgcggttca tgatgtgaca gatgccagcc ccaaccccag ccatgcataa tgtatctgtg

3901 cgccagtgca gccgttatct ccattccgat aacagtgacc agaacaatca gggcattcca

3961 aatccacaac atcgaactta agcaccaggc tgtattcgat tcaccacgcc acagacgcca

4021 ccacggacgc cacggggtca gatgatgttc atggtgacga cccagatgga agcacgtcag

4081 cagagacaga accggaccgt aaccggaact acgtgcgtga tgtgcgtctg caaacggttg

4141 atcggtgtga cgatgcggca gccacgtgcc gaaggtaaac atctgcagtg cgctcagcag

4201 tgccggtgct gcccagaagg tcagcagatt tgccggacgt gcacccagac caaacagtgc

4261 gatcagaacc agagccgtca ggactgccat ttcgcgccag ccgaagtagg tacggaaaaa

4321 gttcaggaac cacggcagaa aagcacgcgg agccggtgca taaaagtccg ggtcatctgc

4381 cgtacccgga gctgcgtgat gagcgtgatg tgcggttttc aggcgatcga aacgaaaacc

4441 ggcatacagg cccagggtca ggcggcccac ggccgcgttc agacgcggac gacccggcgc

4501 cagcgaaccg tgcatggcat catgcgcaac aataaacaga cccactgaca gccacgtctg

4561 cacagcgacg attgccggaa caatcaccag agagctggtg ccccaacgat gaaagtagac

4621 accataaaca tgcagggagc cccagccagc aacaatcata ccagccaggg tcaggccaat

4681 ccaggtttga cgcgggacaa tacgcggttc tgcgactgcg gcggtcatca gcgaagcttt

4741 ttgttctgaa ggagttgtgg tttccttttc ttccacaaca cagatatcgc aaggcaaatg

4801 gcgaccaatc agtttgcggt aggcaaatgc acctacaatc gcgccaacga tagggccgaa

4861 aagcggcacc aggaagtaag gaatgtctct gccgccggta aaggcgacat tgccccagcc

4921 cgccagccag gcaaagactt tcggaccgaa gtcacgcgct gggttcatgg caaaacctgt

4981 caatgggccc atagatgcgc caatgaccgc aatcagtaga ccaatcagca agggagccaa

5041 agggccgcgt ggtacaccgt tgccatcgtc cgttaacgcc aggatcagcc ccatcagaat

5101 agcggtaatc accatctcaa ctgcgaaagc ctgcacaaaa ttgatatgag gattagggta

5161 agtagagaaa gtgccagcca gatcaacact ttcaacgctg ccgcgaacaa tgtgatgagt

5221 ctgctcgaag tcgaaaaata aattgtagta aagcccgtaa actaaagccg cagcacagaa

5281 agcgccggca acttgtgaaa cgataaaagg aataactttg cgcttgtcga aacaggcaaa

5341 cagccacaat gcaatggtaa cagcgggatt aagatgcgcg ccggaaaccc ctgcggtcag

5401 gtagatggcc attgccaccc ccagtcccca aatgacactg atttcccact gaccaaaaga

5461 cgcaccagcg acttttagtg ctgcaacgca acccacaccg aagaaaatca acaacccggt

5521 accgaggaat tcagcaatgc actggccttt caaggttgat gtttgactca tagctgtttc

5581 ctggtttaaa ccgaattggt ggggcgagag gctcaattat atcaacgttg ttatctcttg

5641 tcaacaccgc cagagataa

//

**pPhCCD1**

LOCUS pPhCCD1 4393 bp DNA circular SYN 19-MAR-1980

ACCESSION pPhCCD1

SOURCE Unknown.

ORGANISM Unknown

Unclassified.

REFERENCE 1 (bases 1 to 4393)

AUTHORS Self

JOURNAL Unpublished.

COMMENT SECID/File created by SciEd Central, Scientific & Educational Software

COMMENT This file is created by Vector NTI

http://www.invitrogen.com/

COMMENT ORIGDB|GenBank

COMMENT LSOWNER|

COMMENT VNTNAME|pPhCCD1|

FEATURES Location/Qualifiers

CDS complement (630..1289)

/gene="cat"

/vntifkey="4"

/label=Cm

misc_feature 1653..2563

/vntifkey="21"

/label=p15A

promoter 2589..2744

/vntifkey="30"

/label=M1-46

CDS 2749..4389

/vntifkey="4"

/label=phCCD1

terminator 53..378

/vntifkey="43"

/label=rrnB

BASE COUNT 1133 a 1085 c 1119 g 1056 t

ORIGIN

1 agcctgatac agattaaatc agaacgcaga agcggtctga taaaacagaa tttgcctggc

61 ggcagtagcg cggtggtccc acctgacccc atgccgaact cagaagtgaa acgccgtagc

121 gccgatggta gtgtggggtc tccccatgcg agagtaggga actgccaggc atcaaataaa

181 acgaaaggct cagtcgaaag actgggcctt tcgttttatc tgttgtttgt cggtgaacgc

241 tctcctgagt aggacaaatc cgccgggagc ggatttgaac gttgcgaagc aacggcccgg

301 agggtggcgg gcaggacgcc cgccataaac tgccaggcat caaattaagc agaaggccat

361 cctgacggat ggcctttttg cgtttcttta attaagggag agcctgagca aactggcctc

421 aggcatttga gaagcacacg gtcacactgc ttccggtagt caataaaccg gtaaaccagc

481 aatagacata agcggctatt taacgaccct gccctgaacc gacgaccggg tcgaatttgc

541 tttcgaattt ctgccattca tccgcttatt atcacttatt caggcgtagc accaggcgtt

601 taagggcacc aataactgcc ttaaaaaaat tacgccccgc cctgccactc atcgcagtac

661 tgttgtaatt cattaagcat tctgccgaca tggaagccat cacagacggc atgatgaacc

721 tgaatcgcca gcggcatcag caccttgtcg ccttgcgtat aatatttgcc catggtgaaa

781 acgggggcga agaagttgtc catattggcc acgtttaaat caaaactggt gaaactcacc

841 cagggattgg ctgagacgaa aaacatattc tcaataaacc ctttagggaa ataggccagg

901 ttttcaccgt aacacgccac atcttgcgaa tatatgtgta gaaactgccg gaaatcgtcg

961 tggtattcac tccagagcga tgaaaacgtt tcagtttgct catggaaaac ggtgtaacaa

1021 gggtgaacac tatcccatat caccagctca ccgtctttca ttgccatacg gaattccgga

1081 tgagcattca tcaggcgggc aagaatgtga ataaaggccg gataaaactt gtgcttattt

1141 ttctttacgg tctttaaaaa ggccgtaata tccagctgaa cggtctggtt ataggtacat

1201 tgagcaactg actgaaatgc ctcaaaatgt tctttacgat gccattggga tatatcaacg

1261 gtggtatatc cagtgatttt tttctccatt ttagcttcct tagctcctga aaatctcgat

1321 aactcaaaaa atacgcccgg tagtgatctt atttcattat ggtgaaagtt ggaacctctt

1381 acgtgccgat caacgtctca ttttcgccaa aagttggccc agggcttccc ggtatcaaca

1441 gggacaccag gatttattta ttctgcgaag tgatcttccg tcacaggtat ttattcggcg

1501 caaagtgcgt cgggtgatgc tgccaactta ctgatttagt gtatgatggt gtttttgagg

1561 tgctccagtg gcttctgttt ctatcagctg tccctcctgt tcagctactg acggggtggt

1621 gcgtaacggc aaaagcaccg ccggacatca gcgctagcgg agtgtatact ggcttactat

1681 gttggcactg atgagggtgt cagtgaagtg cttcatgtgg caggagaaaa aaggctgcac

1741 cggtgcgtca gcagaatatg tgatacagga tatattccgc ttcctcgctc actgactcgc

1801 tacgctcggt cgttcgactg cggcgagcgg aaatggctta cgaacggggc ggagatttcc

1861 tggaagatgc caggaagata cttaacaggg aagtgagagg gccgcggcaa agccgttttt

1921 ccataggctc cgcccccctg acaagcatca cgaaatctga cgctcaaatc agtggtggcg

1981 aaacccgaca ggactataaa gataccaggc gtttccccct ggcggctccc tcgtgcgctc

2041 tcctgttcct gcctttcggt ttaccggtgt cattccgctg ttatggccgc gtttgtctca

2101 ttccacgcct gacactcagt tccgggtagg cagttcgctc caagctggac tgtatgcacg

2161 aaccccccgt tcagtccgac cgctgcgcct tatccggtaa ctatcgtctt gagtccaacc

2221 cggaaagaca tgcaaaagca ccactggcag cagccactgg taattgattt agaggagtta

2281 gtcttgaagt catgcgccgg ttaaggctaa actgaaagga caagttttgg tgactgcgct

2341 cctccaagcc agttacctcg gttcaaagag ttggtagctc agagaacctt cgaaaaaccg

2401 ccctgcaagg cggttttttc gttttcagag caagagatta cgcgcagacc aaaacgatct

2461 caagaagatc atcttattaa tcagataaaa tatttctaga tttcagtgca atttatctct

2521 tcaaatgtag cacctgaagt cagccccata cgatataagt tgtaattctc atgtttgaca

2581 gcttgctccg cttggatggc atcctgcctt gttcagaacg ctcggtcttg cacaccgggc

2641 gttttttctt tgtgagtcca ttatctctgg cggtgttgac aagagataac aacgttgata

2701 taattgagcc tctcgcccca ccaattcggt ttaaaccagg aaaccggtat gggtcgtaaa

2761 gaaagcgatg atggcgtgga acgtatcgaa ggcggcgttg tggtggtgaa tccgaagccg

2821 aagaaaggca ttaccgccaa agccatcgac ctgctggaaa aagttattat caaactgatg

2881 cacgatagca gcaaaccgct gcattacctg agcggtaact tcgccccgac agacgaaacc

2941 ccgccgctga acgatctgcc gattaaaggc catctgcctg agtgcctgaa tggcgaattt

3001 gtgcgcgtgg gtccgaaccc gaaatttgcc cctgttgcag gttaccactg gtttgacggc

3061 gatggtatga tccatggtct gcgcatcaag gacggcaaag ccacctatgt tagccgttat

3121 gtgcgcacca gccgcctgaa acaagaagaa tttttcgagg gcgccaagtt catgaaaatt

3181 ggcgacctga agggcttatt cggcctgttt accgtgtaca tgcagatgct gcgcgccaag

3241 ctgaagatcc tggataccag ctacggtaac ggtaccgcca ataccgccct ggtgtatcat

3301 cacggcaaac tgctggccct gagtgaagcc gacaaaccgt acgccctgaa ggtgttagag

3361 gacggtgacc tgcaaaccct gggcatgctg gattacgaca aacgcctgct gcacagtttt

3421 accgcccatc cgaaggttga cccggttacc ggcgagatgt tcacctttgg ttacgcccat

3481 gaaccgccgt atatcaccta ccgcgtgatt agcaaagacg gcattatgca ggatccggtg

3541 ccgatcacca tcccggaagc cattatgatg catgattttg ccattacaga aaattatgcc

3601 attatgatgg atctgccgct gtgctttcgc cctaaggaaa tggtgaaaaa taaccagctg

3661 gcatttacat ttgataccac caaaaaggca cgtttcggcg ttctgccgcg ctatgcaaaa

3721 agcgaagccc tgatccgctg gttcgaactg ccgaactgtt ttatctttca taacgccaac

3781 gcctgggaag aaggtgacga agttgtgctg atcacatgtc gtctgccgca tcctgacctg

3841 gatatggtga atggcgaagt taaagaaaat ctggaaaatt ttagcaatga actgtatgaa

3901 atgcgtttta atatgaaaag cggtgccgcc agccagaaga agctgagcga gagcagcgtt

3961 gatttcccgc gcatcaatga aaactatact ggccgcaagc agcgttacgt gtacggtacc

4021 acactgaaca gcatcgcaaa agtgaccggc atcatcaagt tcgatctgca tgccgagccg

4081 gaaacaggca aaaagcagct ggaagttggt ggcaacgtgc agggcatttt tgacctgggc

4141 cctggccgtt ttggcagcga agcagtgttt gtgccgagcc agccgggcac cgaatgtgaa

4201 gaggatgatg gttacctgat cttttttgtg cacgacgaga acaccggtaa aagcgcagtg

4261 aacgtgattg atgccaaaac catgagtgcc gagccggttg cagtggttga actgccgaag

4321 cgtgtgccgt acggcttcca cgcctttttt gtgacagagg agcagatcca ggagcaggca

4381 aaactgtaac cag

//

**pGlpF-PhCCD1**

LOCUS pGlpF-PhCCD1 5236 bp DNA circular SYN 19-MAR-1980

ACCESSION pGlpF-PhCCD1

SOURCE Unknown.

ORGANISM Unknown

Unclassified.

REFERENCE 1 (bases 1 to 5236)

AUTHORS Self

JOURNAL Unpublished.

COMMENT SECID/File created by SciEd Central, Scientific & Educational Software

COMMENT This file is created by Vector NTI

http://www.invitrogen.com/

COMMENT ORIGDB|GenBank

COMMENT LSOWNER|

COMMENT VNTNAME|pGlpF-PhCCD1|

FEATURES Location/Qualifiers

CDS complement (630..1289)

/gene="cat"

/vntifkey="4"

/label=Cm

misc_feature 1653..2563

/vntifkey="21"

/label=p15A

promoter 2589..2744

/vntifkey="30"

/label=M1-46

CDS 2749..3591

/vntifkey="4"

/label=GlpF

CDS 3592..5232

/vntifkey="4"

/label=phCCD1

terminator 53..378

/vntifkey="43"

/label=rrnB

BASE COUNT 1288 a 1290 c 1345 g 1313 t

ORIGIN

1 agcctgatac agattaaatc agaacgcaga agcggtctga taaaacagaa tttgcctggc

61 ggcagtagcg cggtggtccc acctgacccc atgccgaact cagaagtgaa acgccgtagc

121 gccgatggta gtgtggggtc tccccatgcg agagtaggga actgccaggc atcaaataaa

181 acgaaaggct cagtcgaaag actgggcctt tcgttttatc tgttgtttgt cggtgaacgc

241 tctcctgagt aggacaaatc cgccgggagc ggatttgaac gttgcgaagc aacggcccgg

301 agggtggcgg gcaggacgcc cgccataaac tgccaggcat caaattaagc agaaggccat

361 cctgacggat ggcctttttg cgtttcttta attaagggag agcctgagca aactggcctc

421 aggcatttga gaagcacacg gtcacactgc ttccggtagt caataaaccg gtaaaccagc

481 aatagacata agcggctatt taacgaccct gccctgaacc gacgaccggg tcgaatttgc

541 tttcgaattt ctgccattca tccgcttatt atcacttatt caggcgtagc accaggcgtt

601 taagggcacc aataactgcc ttaaaaaaat tacgccccgc cctgccactc atcgcagtac

661 tgttgtaatt cattaagcat tctgccgaca tggaagccat cacagacggc atgatgaacc

721 tgaatcgcca gcggcatcag caccttgtcg ccttgcgtat aatatttgcc catggtgaaa

781 acgggggcga agaagttgtc catattggcc acgtttaaat caaaactggt gaaactcacc

841 cagggattgg ctgagacgaa aaacatattc tcaataaacc ctttagggaa ataggccagg

901 ttttcaccgt aacacgccac atcttgcgaa tatatgtgta gaaactgccg gaaatcgtcg

961 tggtattcac tccagagcga tgaaaacgtt tcagtttgct catggaaaac ggtgtaacaa

1021 gggtgaacac tatcccatat caccagctca ccgtctttca ttgccatacg gaattccgga

1081 tgagcattca tcaggcgggc aagaatgtga ataaaggccg gataaaactt gtgcttattt

1141 ttctttacgg tctttaaaaa ggccgtaata tccagctgaa cggtctggtt ataggtacat

1201 tgagcaactg actgaaatgc ctcaaaatgt tctttacgat gccattggga tatatcaacg

1261 gtggtatatc cagtgatttt tttctccatt ttagcttcct tagctcctga aaatctcgat

1321 aactcaaaaa atacgcccgg tagtgatctt atttcattat ggtgaaagtt ggaacctctt

1381 acgtgccgat caacgtctca ttttcgccaa aagttggccc agggcttccc ggtatcaaca

1441 gggacaccag gatttattta ttctgcgaag tgatcttccg tcacaggtat ttattcggcg

1501 caaagtgcgt cgggtgatgc tgccaactta ctgatttagt gtatgatggt gtttttgagg

1561 tgctccagtg gcttctgttt ctatcagctg tccctcctgt tcagctactg acggggtggt

1621 gcgtaacggc aaaagcaccg ccggacatca gcgctagcgg agtgtatact ggcttactat

1681 gttggcactg atgagggtgt cagtgaagtg cttcatgtgg caggagaaaa aaggctgcac

1741 cggtgcgtca gcagaatatg tgatacagga tatattccgc ttcctcgctc actgactcgc

1801 tacgctcggt cgttcgactg cggcgagcgg aaatggctta cgaacggggc ggagatttcc

1861 tggaagatgc caggaagata cttaacaggg aagtgagagg gccgcggcaa agccgttttt

1921 ccataggctc cgcccccctg acaagcatca cgaaatctga cgctcaaatc agtggtggcg

1981 aaacccgaca ggactataaa gataccaggc gtttccccct ggcggctccc tcgtgcgctc

2041 tcctgttcct gcctttcggt ttaccggtgt cattccgctg ttatggccgc gtttgtctca

2101 ttccacgcct gacactcagt tccgggtagg cagttcgctc caagctggac tgtatgcacg

2161 aaccccccgt tcagtccgac cgctgcgcct tatccggtaa ctatcgtctt gagtccaacc

2221 cggaaagaca tgcaaaagca ccactggcag cagccactgg taattgattt agaggagtta

2281 gtcttgaagt catgcgccgg ttaaggctaa actgaaagga caagttttgg tgactgcgct

2341 cctccaagcc agttacctcg gttcaaagag ttggtagctc agagaacctt cgaaaaaccg

2401 ccctgcaagg cggttttttc gttttcagag caagagatta cgcgcagacc aaaacgatct

2461 caagaagatc atcttattaa tcagataaaa tatttctaga tttcagtgca atttatctct

2521 tcaaatgtag cacctgaagt cagccccata cgatataagt tgtaattctc atgtttgaca

2581 gcttgctccg cttggatggc atcctgcctt gttcagaacg ctcggtcttg cacaccgggc

2641 gttttttctt tgtgagtcca ttatctctgg cggtgttgac aagagataac aacgttgata

2701 taattgagcc tctcgcccca ccaattcggt ttaaaccagg aaaccggtat gagtcaaaca

2761 tcaaccttga aaggccagtg cattgctgaa ttcctcggta ccgggttgtt gattttcttc

2821 ggtgtgggtt gcgttgcagc actaaaagtc gctggtgcgt cttttggtca gtgggaaatc

2881 agtgtcattt ggggactggg ggtggcaatg gccatctacc tgaccgcagg ggtttccggc

2941 gcgcatctta atcccgctgt taccattgca ttgtggctgt ttgcctgttt cgacaagcgc

3001 aaagttattc cttttatcgt ttcacaagtt gccggcgctt tctgtgctgc ggctttagtt

3061 tacgggcttt actacaattt atttttcgac ttcgagcaga ctcatcacat tgttcgcggc

3121 agcgttgaaa gtgttgatct ggctggcact ttctctactt accctaatcc tcatatcaat

3181 tttgtgcagg ctttcgcagt tgagatggtg attaccgcta ttctgatggg gctgatcctg

3241 gcgttaacgg acgatggcaa cggtgtacca cgcggccctt tggctccctt gctgattggt

3301 ctactgattg cggtcattgg cgcatctatg ggcccattga caggttttgc catgaaccca

3361 gcgcgtgact tcggtccgaa agtctttgcc tggctggcgg gctggggcaa tgtcgccttt

3421 accggcggca gagacattcc ttacttcctg gtgccgcttt tcggccctat cgttggcgcg

3481 attgtaggtg catttgccta ccgcaaactg attggtcgcc atttgccttg cgatatctgt

3541 gttgtggaag aaaaggaaac cacaactcct tcagaacaaa aagcttcgct gatgggtcgt

3601 aaagaaagcg atgatggcgt ggaacgtatc gaaggcggcg ttgtggtggt gaatccgaag

3661 ccgaagaaag gcattaccgc caaagccatc gacctgctgg aaaaagttat tatcaaactg

3721 atgcacgata gcagcaaacc gctgcattac ctgagcggta acttcgcccc gacagacgaa

3781 accccgccgc tgaacgatct gccgattaaa ggccatctgc ctgagtgcct gaatggcgaa

3841 tttgtgcgcg tgggtccgaa cccgaaattt gcccctgttg caggttacca ctggtttgac

3901 ggcgatggta tgatccatgg tctgcgcatc aaggacggca aagccaccta tgttagccgt

3961 tatgtgcgca ccagccgcct gaaacaagaa gaatttttcg agggcgccaa gttcatgaaa

4021 attggcgacc tgaagggctt attcggcctg tttaccgtgt acatgcagat gctgcgcgcc

4081 aagctgaaga tcctggatac cagctacggt aacggtaccg ccaataccgc cctggtgtat

4141 catcacggca aactgctggc cctgagtgaa gccgacaaac cgtacgccct gaaggtgtta

4201 gaggacggtg acctgcaaac cctgggcatg ctggattacg acaaacgcct gctgcacagt

4261 tttaccgccc atccgaaggt tgacccggtt accggcgaga tgttcacctt tggttacgcc

4321 catgaaccgc cgtatatcac ctaccgcgtg attagcaaag acggcattat gcaggatccg

4381 gtgccgatca ccatcccgga agccattatg atgcatgatt ttgccattac agaaaattat

4441 gccattatga tggatctgcc gctgtgcttt cgccctaagg aaatggtgaa aaataaccag

4501 ctggcattta catttgatac caccaaaaag gcacgtttcg gcgttctgcc gcgctatgca

4561 aaaagcgaag ccctgatccg ctggttcgaa ctgccgaact gttttatctt tcataacgcc

4621 aacgcctggg aagaaggtga cgaagttgtg ctgatcacat gtcgtctgcc gcatcctgac

4681 ctggatatgg tgaatggcga agttaaagaa aatctggaaa attttagcaa tgaactgtat

4741 gaaatgcgtt ttaatatgaa aagcggtgcc gccagccaga agaagctgag cgagagcagc

4801 gttgatttcc cgcgcatcaa tgaaaactat actggccgca agcagcgtta cgtgtacggt

4861 accacactga acagcatcgc aaaagtgacc ggcatcatca agttcgatct gcatgccgag

4921 ccggaaacag gcaaaaagca gctggaagtt ggtggcaacg tgcagggcat ttttgacctg

4981 ggccctggcc gttttggcag cgaagcagtg tttgtgccga gccagccggg caccgaatgt

5041 gaagaggatg atggttacct gatctttttt gtgcacgacg agaacaccgg taaaagcgca

5101 gtgaacgtga ttgatgccaa aaccatgagt gccgagccgg ttgcagtggt tgaactgccg

5161 aagcgtgtgc cgtacggctt ccacgccttt tttgtgacag aggagcagat ccaggagcag

5221 gcaaaactgt aaccag

//

**pSPompA-PhCCD1**

LOCUS pSPompA-PhCCD1 4456 bp DNA circular SYN 19-MAR-1980

ACCESSION pSPompA-PhCCD1

SOURCE Unknown.

ORGANISM Unknown

Unclassified.

REFERENCE 1 (bases 1 to 4456)

AUTHORS Self

JOURNAL Unpublished.

COMMENT SECID/File created by SciEd Central, Scientific & Educational Software

COMMENT This file is created by Vector NTI

http://www.invitrogen.com/

COMMENT ORIGDB|GenBank

COMMENT LSOWNER|

COMMENT VNTNAME|pSPompA-PhCCD1|

FEATURES Location/Qualifiers

CDS complement (630..1289)

/gene="cat"

/vntifkey="4"

/label=Cm

misc_feature 1653..2563

/vntifkey="21"

/label=p15A

promoter 2589..2744

/vntifkey="30"

/label=M1-46

sig_peptide 2749..2811

/vntifkey="94"

/label=SP\of\ompA

CDS 2812..4452

/vntifkey="4"

/label=phCCD1

terminator 53..378

/vntifkey="43"

/label=rrnB

BASE COUNT 1148 a 1101 c 1138 g 1069 t

ORIGIN

1 agcctgatac agattaaatc agaacgcaga agcggtctga taaaacagaa tttgcctggc

61 ggcagtagcg cggtggtccc acctgacccc atgccgaact cagaagtgaa acgccgtagc

121 gccgatggta gtgtggggtc tccccatgcg agagtaggga actgccaggc atcaaataaa

181 acgaaaggct cagtcgaaag actgggcctt tcgttttatc tgttgtttgt cggtgaacgc

241 tctcctgagt aggacaaatc cgccgggagc ggatttgaac gttgcgaagc aacggcccgg

301 agggtggcgg gcaggacgcc cgccataaac tgccaggcat caaattaagc agaaggccat

361 cctgacggat ggcctttttg cgtttcttta attaagggag agcctgagca aactggcctc

421 aggcatttga gaagcacacg gtcacactgc ttccggtagt caataaaccg gtaaaccagc

481 aatagacata agcggctatt taacgaccct gccctgaacc gacgaccggg tcgaatttgc

541 tttcgaattt ctgccattca tccgcttatt atcacttatt caggcgtagc accaggcgtt

601 taagggcacc aataactgcc ttaaaaaaat tacgccccgc cctgccactc atcgcagtac

661 tgttgtaatt cattaagcat tctgccgaca tggaagccat cacagacggc atgatgaacc

721 tgaatcgcca gcggcatcag caccttgtcg ccttgcgtat aatatttgcc catggtgaaa

781 acgggggcga agaagttgtc catattggcc acgtttaaat caaaactggt gaaactcacc

841 cagggattgg ctgagacgaa aaacatattc tcaataaacc ctttagggaa ataggccagg

901 ttttcaccgt aacacgccac atcttgcgaa tatatgtgta gaaactgccg gaaatcgtcg

961 tggtattcac tccagagcga tgaaaacgtt tcagtttgct catggaaaac ggtgtaacaa

1021 gggtgaacac tatcccatat caccagctca ccgtctttca ttgccatacg gaattccgga

1081 tgagcattca tcaggcgggc aagaatgtga ataaaggccg gataaaactt gtgcttattt

1141 ttctttacgg tctttaaaaa ggccgtaata tccagctgaa cggtctggtt ataggtacat

1201 tgagcaactg actgaaatgc ctcaaaatgt tctttacgat gccattggga tatatcaacg

1261 gtggtatatc cagtgatttt tttctccatt ttagcttcct tagctcctga aaatctcgat

1321 aactcaaaaa atacgcccgg tagtgatctt atttcattat ggtgaaagtt ggaacctctt

1381 acgtgccgat caacgtctca ttttcgccaa aagttggccc agggcttccc ggtatcaaca

1441 gggacaccag gatttattta ttctgcgaag tgatcttccg tcacaggtat ttattcggcg

1501 caaagtgcgt cgggtgatgc tgccaactta ctgatttagt gtatgatggt gtttttgagg

1561 tgctccagtg gcttctgttt ctatcagctg tccctcctgt tcagctactg acggggtggt

1621 gcgtaacggc aaaagcaccg ccggacatca gcgctagcgg agtgtatact ggcttactat

1681 gttggcactg atgagggtgt cagtgaagtg cttcatgtgg caggagaaaa aaggctgcac

1741 cggtgcgtca gcagaatatg tgatacagga tatattccgc ttcctcgctc actgactcgc

1801 tacgctcggt cgttcgactg cggcgagcgg aaatggctta cgaacggggc ggagatttcc

1861 tggaagatgc caggaagata cttaacaggg aagtgagagg gccgcggcaa agccgttttt

1921 ccataggctc cgcccccctg acaagcatca cgaaatctga cgctcaaatc agtggtggcg

1981 aaacccgaca ggactataaa gataccaggc gtttccccct ggcggctccc tcgtgcgctc

2041 tcctgttcct gcctttcggt ttaccggtgt cattccgctg ttatggccgc gtttgtctca

2101 ttccacgcct gacactcagt tccgggtagg cagttcgctc caagctggac tgtatgcacg

2161 aaccccccgt tcagtccgac cgctgcgcct tatccggtaa ctatcgtctt gagtccaacc

2221 cggaaagaca tgcaaaagca ccactggcag cagccactgg taattgattt agaggagtta

2281 gtcttgaagt catgcgccgg ttaaggctaa actgaaagga caagttttgg tgactgcgct

2341 cctccaagcc agttacctcg gttcaaagag ttggtagctc agagaacctt cgaaaaaccg

2401 ccctgcaagg cggttttttc gttttcagag caagagatta cgcgcagacc aaaacgatct

2461 caagaagatc atcttattaa tcagataaaa tatttctaga tttcagtgca atttatctct

2521 tcaaatgtag cacctgaagt cagccccata cgatataagt tgtaattctc atgtttgaca

2581 gcttgctccg cttggatggc atcctgcctt gttcagaacg ctcggtcttg cacaccgggc

2641 gttttttctt tgtgagtcca ttatctctgg cggtgttgac aagagataac aacgttgata

2701 taattgagcc tctcgcccca ccaattcggt ttaaaccagg aaaccggtat gaaaaagaca

2761 gctatcgcga ttgcagtggc actggctggt ttcgctaccg tagcgcaggc catgggtcgt

2821 aaagaaagcg atgatggcgt ggaacgtatc gaaggcggcg ttgtggtggt gaatccgaag

2881 ccgaagaaag gcattaccgc caaagccatc gacctgctgg aaaaagttat tatcaaactg

2941 atgcacgata gcagcaaacc gctgcattac ctgagcggta acttcgcccc gacagacgaa

3001 accccgccgc tgaacgatct gccgattaaa ggccatctgc ctgagtgcct gaatggcgaa

3061 tttgtgcgcg tgggtccgaa cccgaaattt gcccctgttg caggttacca ctggtttgac

3121 ggcgatggta tgatccatgg tctgcgcatc aaggacggca aagccaccta tgttagccgt

3181 tatgtgcgca ccagccgcct gaaacaagaa gaatttttcg agggcgccaa gttcatgaaa

3241 attggcgacc tgaagggctt attcggcctg tttaccgtgt acatgcagat gctgcgcgcc

3301 aagctgaaga tcctggatac cagctacggt aacggtaccg ccaataccgc cctggtgtat

3361 catcacggca aactgctggc cctgagtgaa gccgacaaac cgtacgccct gaaggtgtta

3421 gaggacggtg acctgcaaac cctgggcatg ctggattacg acaaacgcct gctgcacagt

3481 tttaccgccc atccgaaggt tgacccggtt accggcgaga tgttcacctt tggttacgcc

3541 catgaaccgc cgtatatcac ctaccgcgtg attagcaaag acggcattat gcaggatccg

3601 gtgccgatca ccatcccgga agccattatg atgcatgatt ttgccattac agaaaattat

3661 gccattatga tggatctgcc gctgtgcttt cgccctaagg aaatggtgaa aaataaccag

3721 ctggcattta catttgatac caccaaaaag gcacgtttcg gcgttctgcc gcgctatgca

3781 aaaagcgaag ccctgatccg ctggttcgaa ctgccgaact gttttatctt tcataacgcc

3841 aacgcctggg aagaaggtga cgaagttgtg ctgatcacat gtcgtctgcc gcatcctgac

3901 ctggatatgg tgaatggcga agttaaagaa aatctggaaa attttagcaa tgaactgtat

3961 gaaatgcgtt ttaatatgaa aagcggtgcc gccagccaga agaagctgag cgagagcagc

4021 gttgatttcc cgcgcatcaa tgaaaactat actggccgca agcagcgtta cgtgtacggt

4081 accacactga acagcatcgc aaaagtgacc ggcatcatca agttcgatct gcatgccgag

4141 ccggaaacag gcaaaaagca gctggaagtt ggtggcaacg tgcagggcat ttttgacctg

4201 ggccctggcc gttttggcag cgaagcagtg tttgtgccga gccagccggg caccgaatgt

4261 gaagaggatg atggttacct gatctttttt gtgcacgacg agaacaccgg taaaagcgca

4321 gtgaacgtga ttgatgccaa aaccatgagt gccgagccgg ttgcagtggt tgaactgccg

4381 aagcgtgtgc cgtacggctt ccacgccttt tttgtgacag aggagcagat ccaggagcag

4441 gcaaaactgt aaccag

//

**pMBP-PhCCD1**

LOCUS pMBP-PhCCD1 5494 bp DNA circular SYN 19-MAR-1980

ACCESSION pMBP-PhCCD1

SOURCE Unknown.

ORGANISM Unknown

Unclassified.

REFERENCE 1 (bases 1 to 5494)

AUTHORS Self

JOURNAL Unpublished.

COMMENT SECID/File created by SciEd Central, Scientific & Educational Software

COMMENT This file is created by Vector NTI

http://www.invitrogen.com/

COMMENT ORIGDB|GenBank

COMMENT LSOWNER|

COMMENT VNTNAME|pMBP-PhCCD1|

FEATURES Location/Qualifiers

CDS complement (630..1289)

/gene="cat"

/vntifkey="4"

/label=Cm

misc_feature 1653..2563

/vntifkey="21"

/label=p15A

promoter 2589..2744

/vntifkey="30"

/label=M1-46

CDS 2749..3849

/vntifkey="4"

/label=MBP

CDS 3850..5490

/vntifkey="4"

/label=phCCD1

terminator 53..378

/vntifkey="43"

/label=rrnB

BASE COUNT 1447 a 1354 c 1414 g 1279 t

ORIGIN

1 agcctgatac agattaaatc agaacgcaga agcggtctga taaaacagaa tttgcctggc

61 ggcagtagcg cggtggtccc acctgacccc atgccgaact cagaagtgaa acgccgtagc

121 gccgatggta gtgtggggtc tccccatgcg agagtaggga actgccaggc atcaaataaa

181 acgaaaggct cagtcgaaag actgggcctt tcgttttatc tgttgtttgt cggtgaacgc

241 tctcctgagt aggacaaatc cgccgggagc ggatttgaac gttgcgaagc aacggcccgg

301 agggtggcgg gcaggacgcc cgccataaac tgccaggcat caaattaagc agaaggccat

361 cctgacggat ggcctttttg cgtttcttta attaagggag agcctgagca aactggcctc

421 aggcatttga gaagcacacg gtcacactgc ttccggtagt caataaaccg gtaaaccagc

481 aatagacata agcggctatt taacgaccct gccctgaacc gacgaccggg tcgaatttgc

541 tttcgaattt ctgccattca tccgcttatt atcacttatt caggcgtagc accaggcgtt

601 taagggcacc aataactgcc ttaaaaaaat tacgccccgc cctgccactc atcgcagtac

661 tgttgtaatt cattaagcat tctgccgaca tggaagccat cacagacggc atgatgaacc

721 tgaatcgcca gcggcatcag caccttgtcg ccttgcgtat aatatttgcc catggtgaaa

781 acgggggcga agaagttgtc catattggcc acgtttaaat caaaactggt gaaactcacc

841 cagggattgg ctgagacgaa aaacatattc tcaataaacc ctttagggaa ataggccagg

901 ttttcaccgt aacacgccac atcttgcgaa tatatgtgta gaaactgccg gaaatcgtcg

961 tggtattcac tccagagcga tgaaaacgtt tcagtttgct catggaaaac ggtgtaacaa

1021 gggtgaacac tatcccatat caccagctca ccgtctttca ttgccatacg gaattccgga

1081 tgagcattca tcaggcgggc aagaatgtga ataaaggccg gataaaactt gtgcttattt

1141 ttctttacgg tctttaaaaa ggccgtaata tccagctgaa cggtctggtt ataggtacat

1201 tgagcaactg actgaaatgc ctcaaaatgt tctttacgat gccattggga tatatcaacg

1261 gtggtatatc cagtgatttt tttctccatt ttagcttcct tagctcctga aaatctcgat

1321 aactcaaaaa atacgcccgg tagtgatctt atttcattat ggtgaaagtt ggaacctctt

1381 acgtgccgat caacgtctca ttttcgccaa aagttggccc agggcttccc ggtatcaaca

1441 gggacaccag gatttattta ttctgcgaag tgatcttccg tcacaggtat ttattcggcg

1501 caaagtgcgt cgggtgatgc tgccaactta ctgatttagt gtatgatggt gtttttgagg

1561 tgctccagtg gcttctgttt ctatcagctg tccctcctgt tcagctactg acggggtggt

1621 gcgtaacggc aaaagcaccg ccggacatca gcgctagcgg agtgtatact ggcttactat

1681 gttggcactg atgagggtgt cagtgaagtg cttcatgtgg caggagaaaa aaggctgcac

1741 cggtgcgtca gcagaatatg tgatacagga tatattccgc ttcctcgctc actgactcgc

1801 tacgctcggt cgttcgactg cggcgagcgg aaatggctta cgaacggggc ggagatttcc

1861 tggaagatgc caggaagata cttaacaggg aagtgagagg gccgcggcaa agccgttttt

1921 ccataggctc cgcccccctg acaagcatca cgaaatctga cgctcaaatc agtggtggcg

1981 aaacccgaca ggactataaa gataccaggc gtttccccct ggcggctccc tcgtgcgctc

2041 tcctgttcct gcctttcggt ttaccggtgt cattccgctg ttatggccgc gtttgtctca

2101 ttccacgcct gacactcagt tccgggtagg cagttcgctc caagctggac tgtatgcacg

2161 aaccccccgt tcagtccgac cgctgcgcct tatccggtaa ctatcgtctt gagtccaacc

2221 cggaaagaca tgcaaaagca ccactggcag cagccactgg taattgattt agaggagtta

2281 gtcttgaagt catgcgccgg ttaaggctaa actgaaagga caagttttgg tgactgcgct

2341 cctccaagcc agttacctcg gttcaaagag ttggtagctc agagaacctt cgaaaaaccg

2401 ccctgcaagg cggttttttc gttttcagag caagagatta cgcgcagacc aaaacgatct

2461 caagaagatc atcttattaa tcagataaaa tatttctaga tttcagtgca atttatctct

2521 tcaaatgtag cacctgaagt cagccccata cgatataagt tgtaattctc atgtttgaca

2581 gcttgctccg cttggatggc atcctgcctt gttcagaacg ctcggtcttg cacaccgggc

2641 gttttttctt tgtgagtcca ttatctctgg cggtgttgac aagagataac aacgttgata

2701 taattgagcc tctcgcccca ccaattcggt ttaaaccagg aaaccggtat gaaaatcgaa

2761 gaaggtaaac tggtaatctg gattaacggc gataaaggct ataacggtct cgctgaagtc

2821 ggtaagaaat tcgagaaaga taccggaatt aaagtcaccg ttgagcatcc ggataaactg

2881 gaagagaaat tcccacaggt tgcggcaact ggcgatggcc ctgacattat cttctgggca

2941 cacgaccgct ttggtggcta cgctcaatct ggcctgttgg ctgaaatcac cccggacaaa

3001 gcgttccagg acaagctgta tccgtttacc tgggatgccg tacgttacaa cggcaagctg

3061 attgcttacc cgatcgctgt tgaagcgtta tcgctgattt ataacaaaga tctgctgccg

3121 aacccgccaa aaacctggga agagatcccg gcgctggata aagaactgaa agcgaaaggt

3181 aagagcgcgc tgatgttcaa cctgcaagaa ccgtacttca cctggccgct gattgctgct

3241 gacgggggtt atgcgttcaa gtatgaaaac ggcaagtaca acattaaaga cgtgggcgtg

3301 gataacgctg gcgcgaaagc gggtctgacc ttcctggttg acctgattaa aaacaaacac

3361 atgaatgcag acaccgatta ctccatcgca gaagctgcct ttaataaagg cgaaacagcg

3421 atgaccatca acggcccgtg ggcatggtcc aacatcgaca ccagcaaagt gaattatggt

3481 gtaacggtac tgccgacctt caagggtcaa ccatccaaac cgttcgttgg cgtgctgagc

3541 gcaggtatta acgccgccag tccgaacaaa gagctggcga aagagttcct cgaaaactat

3601 ctgctgactg atgaaggtct ggaagcggtt aataaagaca aaccgctggg tgccgtagcg

3661 ctgaagtctt acgaggaaga gttggcgaaa gatccacgta ttgccgccac catggaaaac

3721 gcccagaaag gtgaaatcat gccgaacatc ccgcagatgt ccgctttctg gtatgccgtg

3781 cgtactgcgg tgatcaacgc cgccagcggt cgtcagactg tcgatgaagc cctgaaagac

3841 gcgcagacta tgggtcgtaa agaaagcgat gatggcgtgg aacgtatcga aggcggcgtt

3901 gtggtggtga atccgaagcc gaagaaaggc attaccgcca aagccatcga cctgctggaa

3961 aaagttatta tcaaactgat gcacgatagc agcaaaccgc tgcattacct gagcggtaac

4021 ttcgccccga cagacgaaac cccgccgctg aacgatctgc cgattaaagg ccatctgcct

4081 gagtgcctga atggcgaatt tgtgcgcgtg ggtccgaacc cgaaatttgc ccctgttgca

4141 ggttaccact ggtttgacgg cgatggtatg atccatggtc tgcgcatcaa ggacggcaaa

4201 gccacctatg ttagccgtta tgtgcgcacc agccgcctga aacaagaaga atttttcgag

4261 ggcgccaagt tcatgaaaat tggcgacctg aagggcttat tcggcctgtt taccgtgtac

4321 atgcagatgc tgcgcgccaa gctgaagatc ctggatacca gctacggtaa cggtaccgcc

4381 aataccgccc tggtgtatca tcacggcaaa ctgctggccc tgagtgaagc cgacaaaccg

4441 tacgccctga aggtgttaga ggacggtgac ctgcaaaccc tgggcatgct ggattacgac

4501 aaacgcctgc tgcacagttt taccgcccat ccgaaggttg acccggttac cggcgagatg

4561 ttcacctttg gttacgccca tgaaccgccg tatatcacct accgcgtgat tagcaaagac

4621 ggcattatgc aggatccggt gccgatcacc atcccggaag ccattatgat gcatgatttt

4681 gccattacag aaaattatgc cattatgatg gatctgccgc tgtgctttcg ccctaaggaa

4741 atggtgaaaa ataaccagct ggcatttaca tttgatacca ccaaaaaggc acgtttcggc

4801 gttctgccgc gctatgcaaa aagcgaagcc ctgatccgct ggttcgaact gccgaactgt

4861 tttatctttc ataacgccaa cgcctgggaa gaaggtgacg aagttgtgct gatcacatgt

4921 cgtctgccgc atcctgacct ggatatggtg aatggcgaag ttaaagaaaa tctggaaaat

4981 tttagcaatg aactgtatga aatgcgtttt aatatgaaaa gcggtgccgc cagccagaag

5041 aagctgagcg agagcagcgt tgatttcccg cgcatcaatg aaaactatac tggccgcaag

5101 cagcgttacg tgtacggtac cacactgaac agcatcgcaa aagtgaccgg catcatcaag

5161 ttcgatctgc atgccgagcc ggaaacaggc aaaaagcagc tggaagttgg tggcaacgtg

5221 cagggcattt ttgacctggg ccctggccgt tttggcagcg aagcagtgtt tgtgccgagc

5281 cagccgggca ccgaatgtga agaggatgat ggttacctga tcttttttgt gcacgacgag

5341 aacaccggta aaagcgcagt gaacgtgatt gatgccaaaa ccatgagtgc cgagccggtt

5401 gcagtggttg aactgccgaa gcgtgtgccg tacggcttcc acgccttttt tgtgacagag

5461 gagcagatcc aggagcaggc aaaactgtaa ccag

//
